# Changes in the genetic structure of Atlantic salmon populations over four decades reveal substantial impacts of stocking and potential resiliency

**DOI:** 10.1002/ece3.629

**Published:** 2013-06-12

**Authors:** Charles Perrier, René Guyomard, Jean-Luc Bagliniere, Natacha Nikolic, Guillaume Evanno

**Affiliations:** 1 Institut de Biologie Intégrative et des Systèmes (IBIS), Université LavalQuébec, Canada; 2 INRA, UMR 0985 Ecology and Health of Ecosystems35042, Rennes, France; 3 Agrocampus Ouest65 rue de Saint-Brieuc, 35042, Rennes, France; 4 INRA, UMR 1313 Génétique Animale et Biologie IntégrativeF-78350, Jouy-en-Josas, France; 5 IFREMERDélégation de La Réunion, 97822 Le Port Cedex, La Réunion, France

**Keywords:** Conservation, population genetics, *Salmo salar*, stocking, temporal stability

## Abstract

While the stocking of captive-bred fish has been occurring for decades and has had substantial immediate genetic and evolutionary impacts on wild populations, its long-term consequences have only been weakly investigated. Here, we conducted a spatiotemporal analysis of 1428 Atlantic salmon sampled from 1965 to 2006 in 25 populations throughout France to investigate the influence of stocking on the neutral genetic structure in wild Atlantic salmon (*Salmo salar*) populations. On the basis of the analysis of 11 microsatellite loci, we found that the overall genetic structure among populations dramatically decreased over the period studied. Admixture rates among populations were highly variable, ranging from a nearly undetectable contribution from donor stocks to total replacement of the native gene pool, suggesting extremely variable impacts of stocking. Depending on population, admixture rates either increased, remained stable, or decreased in samples collected between 1998 and 2006 compared to samples from 1965 to 1987, suggesting either rising, long-lasting or short-term impacts of stocking. We discuss the potential mechanisms contributing to this variability, including the reduced fitness of stocked fish and persistence of wild locally adapted individuals.

## Introduction

Anthropogenic pressures on natural populations have the potential to alter the distribution of genetic diversity within and among wild populations. By comparing historical and current genetic diversity we can test for changes in the genetic structure of populations as a result of such perturbations (Schwartz et al. 2007; Nielsen and Hansen 2008). For instance, genetic information from archived fish scales can allow for inferences to be made about the recent evolution of genetic structure among wild fish populations (Nielsen et al. 1999; Nielsen and Hansen 2008).

The neutral genetic structure among numerous Salmonid populations is thought to have been recently affected by various increasing anthropogenic pressures such as rapid contemporary climate change, habitat degradation and disconnection, fish farm escapees, and stocking. Global warming may have affected gene flow among populations (Valiente et al. 2010; Horreo et al. 2011) as well as habitat connectivity (Neraas and Spruell 2001; Kanno et al. 2011). Farmed escapees are also responsible for low to modest modifications of population genetic diversity in relation to the density of native populations (Glover et al. 2012). However, one of the most widespread and rapid ways of modification in the genetic structure within and among wild fish populations may occur through the translocation of hatchery-reared individuals. Such transfers of fish may substantially increase the local neutral genetic diversity (Marie et al. 2010) and lead to variable admixture rates between target and source populations (Campos et al. 2008; Finnengan and Stevens 2008; Sonstebo et al. 2008; Hansen et al. 2009; Perrier et al. 2011b). Moreover, major decreases in population differentiation have been observed among stocked populations and/or among sources and targets of stocking (Finnengan and Stevens 2008; Eldridge et al. 2009; Marie et al. 2010; Perrier et al. 2011b). Hatchery-reared fish also can have high dispersal rates (Quinn 1993; Jonsson et al. 2003), which may lead to large-scale impacts of stocking in nontargeted populations (Perrier et al. 2011b, 2013). Finally, stocking may also disrupt the relationship between population genetic structure and its environmental determinants, causing the erosion of original patterns of isolation by distance (Eldridge and Naish 2007; Pearse et al. 2011; Perrier et al. 2011b).

In addition to changing the distribution of neutral genetic diversity within and among wild populations, such supplementation practices may alter the fitness of Salmonid populations via introgressive hybridization (Levin et al. 2001; Aprahamian et al. 2003; Araki and Schmid 2010). Indeed, captive breeding may select for traits that are disadvantageous in the wild (Blanchet et al. 2008; Fraser 2008; Williams and Hoffman 2009). As salmonid populations are often locally adapted (Garcia de Leaniz et al. 2007; Fraser et al. 2011), the genetic admixture between wild and hatchery and/or nonnative individuals may ultimately result in a loss of local adaptation and reduced fitness in wild populations (McGinnity et al. 2003; Araki et al. 2007; Ford and Myers 2008; Milot et al. 2013; but see Fitzpatrick et al. 2010).

While high impacts of stocking practices on neutral population genetic structure have been widely documented, there are poor evidences and understanding of remarkably low impacts and/or short-term incidences of stocking programs (Santos et al. 2006; Finnengan and Stevens 2008; Caudron et al. 2009; Eldridge et al. 2009; Hansen and Mensberg 2009; Gow et al. 2011). In particular, the too rare analysis of historical samples precludes the possibility of documenting short-term admixture. Hence, it is crucial to document and understand situations where stocking may not always lead to high and/or long-lasting changes in neutral genetic structure of stocked populations. Such relatively low effects of stocking may be the result of various mechanisms leading to lower survival and reproduction of stocked individuals in their new habitat. For example, low performances of captive-bred fish released in natura (Fleming and Einum 1997; Araki et al. 2009; Thériault et al. 2011; Milot et al. 2013) relative to their wild, locally adapted conspecifics may lead to a low introgression and a rapid recovery of genetic structure. Furthermore, some reproductive isolation may occur as a result of either isolation by time or space, and may also contribute to the persistence of indigenous individuals (Hansen and Mensberg 2009) or subpopulations (Eldridge et al. 2009). The relative abundance of stocked and wild individuals may also be a determinant factor in the process of introgression; that is, much lower abundances of stocked fish compared to wild individuals can clearly lead to weak introgression (Currat et al. 2008; Perrier et al. 2013). Finally, dispersal and/or recolonization by nearby wild populations (Vasemagi et al. 2001; Perrier et al. 2010, 2013) may further dilute the local impacts of stocking. Intense but ancient stocking practices can thus have relatively weak and short-term impacts on the genetic makeup of targeted populations. However, investigating this hypothesis requires temporal samples collected before, during, and after stocking events to estimate the immediate impacts of such practices and their long-term persistence.

In this study, we investigated the genetic effects of a variety of stocking strategies using Atlantic salmon populations from France as a case study. Some of these populations were stocked from 1950s to 1980s using nonlocal fish (mainly from Scotland) (Baglinière and Dumas 1988; Baglinière et al. 1990; Vauclin 2007), while other populations have only been stocked since the 1990s using local fish or nonnative but “French” salmon (Vauclin 2007; Grandjean et al. 2009). Thanks to scientific and management programs initiated in the 1960s salmon scales have been continuously collected in most French rivers, allowing for temporal analyses of genetic structure. Although several populations have been found to be moderately to highly admixed following stocking, no admixture was detected in present day samples from several French populations stocked during 1960s–1980s (Perrier et al. 2011a,b). Therefore, samples collected before and during nonnative stocking operations are needed to thoroughly investigate the potential short-term impacts of stocking on the neutral genetic structure of populations. In other populations, samples collected at the time of, and several generations after, stocking constitute a unique opportunity to explore the long-term impacts of stocking. These impacts may be persistent if replacement of the local gene pool occurs, but could also be temporary if there is a reduced fitness of stocked fish relative to wild individuals. Therefore, depending on the population, time series of samples were either used to study impacts of recent stocking or the long-term impacts of previous supplementation practices. We analyzed 11 microsatellite loci in historical (1965–1989 cohorts) and recent samples (1998–2006) from 25 Atlantic salmon (*Salmo salar*) populations in France and two Scottish populations. We tested whether stocking changed the genetic admixture rates within salmon populations and led to a reduction in genetic differentiation among donor and targeted populations. We also examined if such practices may have led to a decrease in the overall genetic structure among populations in France. We then investigated if such practices may have only short-term effects on the neutral genetic diversity of wild populations by comparing the level of genetic diversity in some populations at the time of stocking and several generations after the end of these operations. We hypothesized that stocking may have mixed effects on genetic diversity including (i) a global homogenization of genetic diversity among populations, (ii) variable admixture rates of stocked populations and a decrease in differentiation between donors and targets of stocking, and (iii) only short-term changes in genetic variation.

## Materials and Methods

### Study populations and sampling

**Figure 1:**
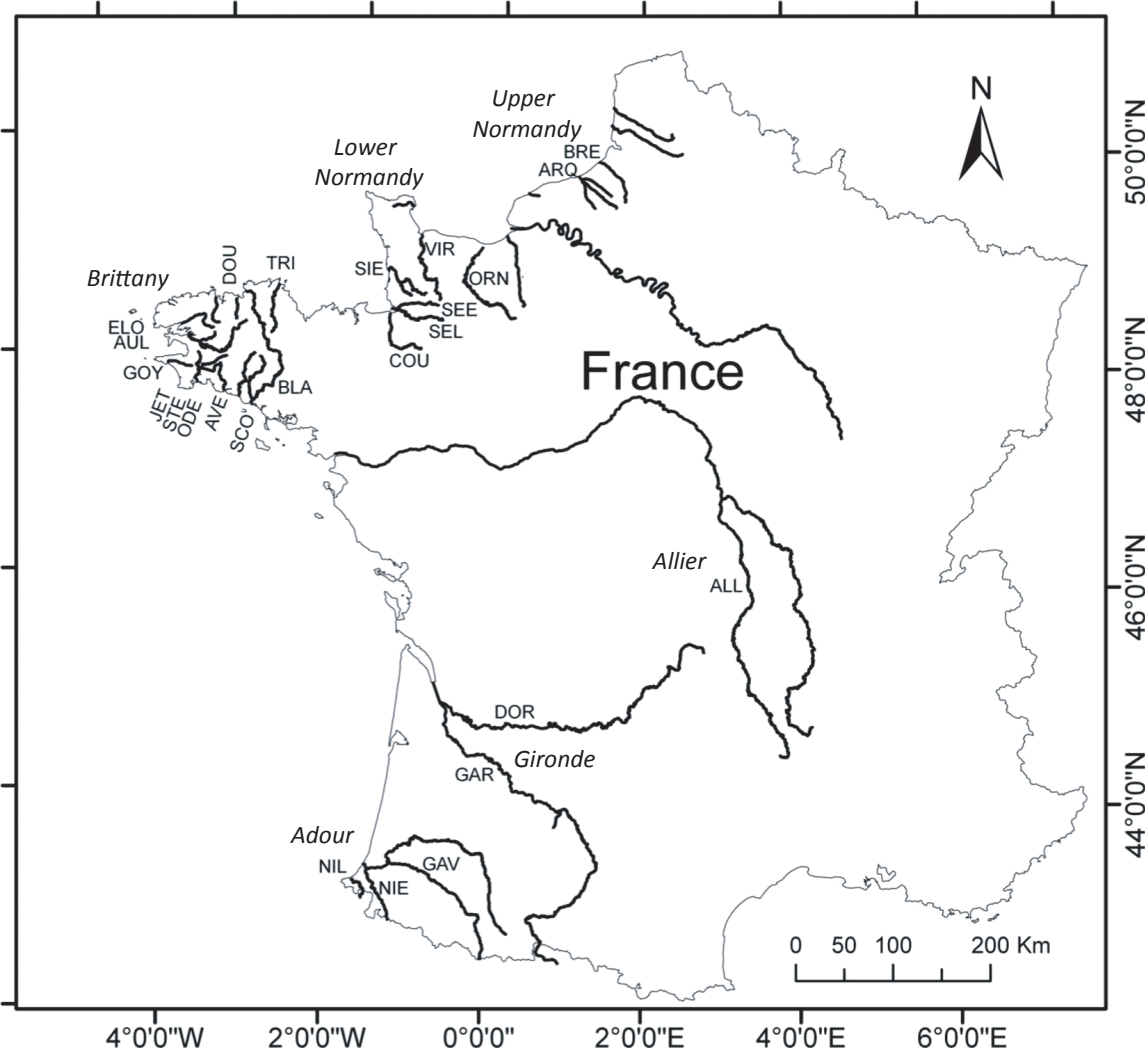
Map showing locations of the study populations (see also Table 1).

We studied 25 Atlantic salmon populations from France and two from Scotland (Fig. 1, Table 1). We collected samples from cohorts from 1965 to 1989 and cohorts from 1998 to 2006. Data from recent cohorts (1998–2006) have been previously used by Perrier et al. (2011b). As we could not genotype individuals from the main Scottish source populations (Dee, Tay, and Thurso rivers), we used samples from the proximal Spey and Shin rivers, which have been analyzed by Nikolic et al. (2009a,b). These Scottish populations appear to be genetically similar and belong to the same genetic cluster of northern Scotland and Ireland, which is well differentiated from French populations (Griffiths et al. 2010; J. Gilbey pers. comm.). Adult fish from French populations were collected by angling or trapping and scales were stored by INRA (French National Institute for Agronomic Research) and ONEMA (French National Agency for Water and Aquatic Environments) in 95% ethanol or in paper envelopes. The age (number of years in river + number of years at sea) of each individual was determined from scale growth patterns (Lund and Hansel 1991; Stokesbury and Lacroix 1997).

**Table 1:** Description of populations' characteristics and sampling for 25 sampled French rivers and two Scottish ones

Region	River	Historical cohorts				Recent cohorts			
	
		Abbreviation	Cohorts	Sample size	Stocking from 1950 to 1988	Abbreviation	Cohorts	Sample size	Stocking from 1989 to 2006
Scotland	Spey	H-SPE	1985	48	–	R-SPE	2003	40	–
	Shin	H-SHI	1989	48	–	R-SHI	2003	41	–
Upper Normandy	Bresle	H-BRE	1968	19	Scotland, Scandinavia	R-BRE	1998–2004	29	–
	Arques	–	–	–	Scotland, Scandinavia	R-ARQ	2003	31	–
Lower Normandy	Orne	–	–	–	Scotland, Sélune	R-ORN	2001	31	Gave d'Oloron
	Vire	–	–	–	Scotland	R-VIR	1998–2004	19	–
	Sienne	H-SIE	1985–1987	35	–	R-SIE	2002–2003	36	Aulne
	Sée	H-SEE	1977–1978	61	–	R-SEE	2002–2003	66	Aulne
	Sélune	H-SEL	1977–1978	39	–	R-SEL	2002–2003	80	Aulne and Gave d'Oloron
	Couesnon	H-COU	1978–1986	11	Sélune	R-COU	2002–2003	34	Aulne and Gave d'Oloron
Brittany	Trieux	H-TRI	1968–1981	17	Scotland	R-TRI	2002	16	–
	Douron	H-DOU	1978–1984	29	Scotland	R-DOU	2002–2003	27	–
	Elorn	H-ELO	1969–1970	18	Scotland, local	R-ELO	2003	30	Local
	Aulne	H-AUL	1969–1984	30	Scotland, local	R-AUL	2003	31	Local
	Goyen	H-GOY	1972–1984	33	–	R-GOY	2003	24	–
	Steir	H-STE	1971–1972	21	Scotland	R-STE	2002	14	–
	Jet	H-JET	1971–1973	11	Scotland	R-JET	2000–2004	17	–
	Odet	H-ODE	1972–1973	19	Scotland	R-ODE	2003	14	–
	Aven	H-AVE	1973–1978	40	Local	R-AVE	2003	34	–
	Scorff	H-SCO	1977–1978	64	Scotland, local	R-SCO	2002–2003	64	–
	Blavet	H-BLA	1977–1978	65	Scotland, local	R-BLA	2002–2003	63	–
Allier	Allier	H-ALL	1965–1967	49	Gave d'Oloron, Scotland, Canada, Scandinavia, local	R-ALL	2001–2002	31	Local
Gironde	Dordogne	–	–	–	Scotland, Allier, Gave d'Oloron, local	R-DOR	2002	15	Local
	Garonne	–	–	–	Scotland, Allier, Gave d'Oloron, local	R-GAR	2002	30	Local
Adour	Gave d'Oloron	H-GAV	1984–1984	25	Scotland, Scandinavia, local	R-GAV	2003	29	Local
	Nive	H-NIE	1984–1984	26	Scotland, local	R-NIE	2001–2006	8	–
	Nivelle	H-NIL	1977–1987	26	Scotland, local	R-NIL	1998–2004	17	–

Supplementation operations occurred in many different rivers at different times. Before 1990, French stocking programs mainly used nonlocal fish originating from Scotland (Spey, Conon, Dee, Tay, and Thurso rivers), but some rivers were stocked with fish from different French rivers (Baglinière and Dumas 1988; Baglinière et al. 1990; Table 1). Since 1990, the use of fish from other countries has been prohibited and stocking with individuals that are native to the stocked river has become the rule. However, some rivers have been stocked with other French populations (Perrier et al. 2011b).

### Molecular analyses

Genomic DNA was extracted from *S. salar* scales by heating samples in a solution of proteinase K, TE (Tris/EDTA) buffer and chelex, at 55°C 2 h and then at 100°C for 10 min (Estoup et al. 1996). The M13 method (Schuelke 2000) was used to label DNA polymerase chain reaction (PCR) amplifications. We used 11 microsatellites: BHMS176; BHMS429; SSA85; SSO SL85; BHMS235; BHMS111; SSA197; SSA171; BHMS 377; SSSP2216; SSA224. Details on markers and PCR conditions are given in Nikolic et al. (2009b). Finally, some samples from French populations (*n* = 395, 28% of total) were analyzed at the SSAD486 locus in order to document if brown trout or hybrids between *S. salar* and *S. trutta* were misidentified as salmon (Perrier et al. 2011a). Misidentified fish were discarded from subsequent analyses. Fragment analysis was achieved using Genemapper 4.0 software (Applied Biosystems, Foster City, CA).

### Data analyses

We used MICRO-CHECKER 2.2.3 (van Oosterhout et al. 2004) to assess the frequency of null alleles and scoring errors due to stuttering or large allelic dropout. Allele number and allelic richness were obtained using FSTAT 2.9.3.2 (Goudet 1995). Tests for linkage and Hardy–Weinberg disequilibria were conducted with FSTAT 2.9.3.2. Expected heterozygosity, *H*_e_, (Nei 1978) and observed heterozygosity, *H*_o_, were calculated with GENETIX 4.05.2 (Belkhir et al. 1996).

The VAREFF software, implemented as an R package (http://cran.r-project.org/web/packages/VarEff), was used to infer effective size of populations and their recent evolution. The function theta was used to obtain an overview of the current and ancestral average of *θ* (4 × *N*_e_ × *μ*) with *N*_e_ the effective size and *μ* the mutation rate. The function VarEff was then used to characterize effective population sizes. We used a stepwise mutation model for microsatellites with *μ* = 6 × 10^−4^ (Nikolic et al. 2009a). Results were based on Markov Chain Monte Carlo (MCMC) chains including 10^4^ dememorization steps, a total length of 10^6^ and the extraction of 10^4^ uncorrelated states (Chevalet and Nikolic 2010).

We used the Bayesian individual clustering approach implemented in Structure to determine the hierarchical genetic structure of the study populations and the admixture rates of stocked populations (Pritchard et al. 2000). We ran six different analyses all assuming an admixture model (i.e., allowing the genetic composition of individuals to be a mixture from different populations). For each analysis, we tested from 1 to *n* + 1 genetic clusters (*k*) with n equal to the number of putative populations, and performed 15 replicates for each *k*. Each run started with a burn-in period of 50,000 steps followed by 300,000 MCMC replicates. For each individual, the 90% credible intervals of admixture values were estimated. We selected the *k* with the highest likelihood (Pritchard et al. 2000) and according to the *Δk* method (Evanno et al. 2005). The first analysis was performed using all samples in order to determine the hierarchical genetic structure among populations and to gain a coarse estimate of admixture. We then performed five restricted analyses each including a subset of populations (groups of targeted and donor populations) to decrease statistical noise and the uncertainty associated with the increase in the number of clusters and to refine admixture estimates.

Pairwise population *F*_st_'s were computed in FSTAT 2.9.3.2 and Global *F*_st_ estimates were calculated with GENETIX 4.05.2. A neighbor-joining dendrogram based on pairwise Nei (Da) genetic distances (Nei et al. 1983) was built using MEGA4 (Tamura et al. 2007). Analyses of molecular variance (AMOVA) were performed using ARLEQUIN (Excoffier et al. 2005). The hierarchical grouping of populations used for AMOVAs was defined according to the neighbor-joining dendrogram and Bayesian clustering analyses with Structure (Pritchard et al. 2000). Temporal pairwise *F*_st_'s were computed for each river with GENETIX 4.05.2 (Belkhir et al. 1996).

## Results

### Genetic diversity and effective population size

Multilocus genotype information was obtained for 1434 individuals from France and 177 individuals from Scotland (Table 1). Amplification success was generally high, ranging from 91.6% to 99.9% depending on the sample, and from 89% to 99.9% depending on the locus. It was of 95.1% for historical samples and of 98.1% for recent samples with an overall rate of 96.5% amplification success. Among samples analyzed at the SSAD486 locus, five (1.2%) individuals were identified as brown trout and one (0.2%) individual as a hybrid between *S. salar* and *S. trutta* that may have been misidentified as salmon. These six misidentified individuals were discarded and subsequent analyses were conducted on 1428 individuals. A total of 27 of the 495 *F*_IS_ values were significantly different from zero. MICRO-CHECKER did not detect any evidence of large allelic dropout or stuttering. However, it suggested the presence of 15 null alleles among the 495 tests performed. Only two of these indications of null alleles were associated with an *F*_IS_ significantly different from zero.

**Figure 2:**
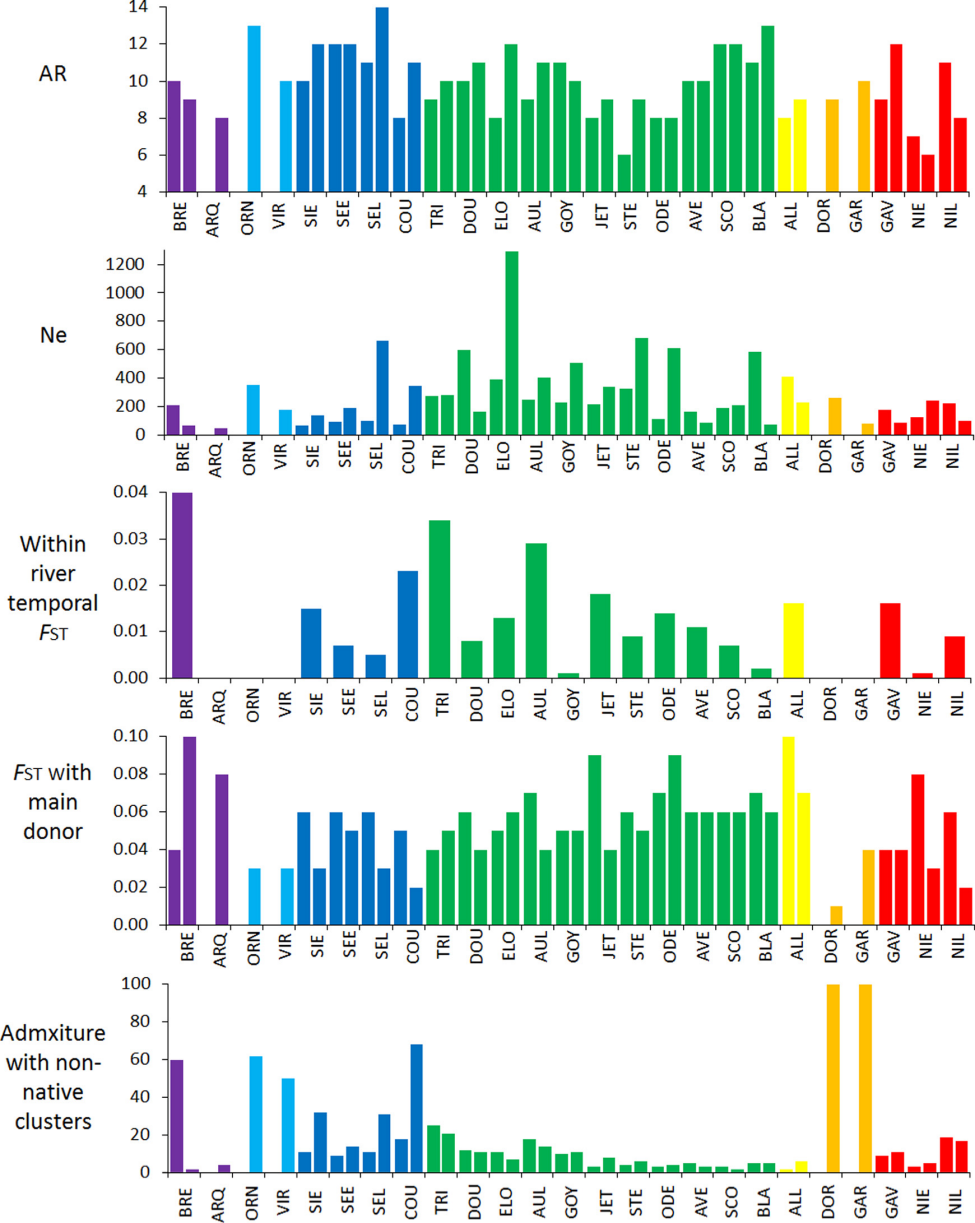
Allelic richness (AR), effective size (*N*_e_), within river *F*_st_, *F*_st_ between the considered river and the main donor stock, and population admixture proportion with nonlocal clusters in Atlantic salmon samples from French populations. Left and right bars correspond, respectively, to the historical and recent samples for each populations except for within river *F*_st_ (one single bar) and for Arques (ARQ), Orne (ORN), Vire (VIR), Dordogne (DOR), Garonne (GAR) (only recent samples).

Average expected heterozygosity was 0.80 among samples from French populations and ranged from 0.74 to 0.85 (Table S1). Average allelic richness was 10 and ranged from 6 to 14 (Table 2 and Fig. 2). Within the Bresle population, average allelic richness over all loci were 10 and 9 at the time of, and after stocking, respectively. Within Lower Normandy populations, average allelic richness was 10.2 (8–12) and 12.3 (11–14) before and at the time of stocking, respectively. Within populations from Brittany, average allelic richness was 9.3 and 10.5 at the time of, and after stocking, respectively. Within the Allier population allelic richness was 8 and 9 at the time of, and after stocking, respectively. Within Adour populations, allelic richness was on average 9.0 and 8.7 contemporary to and after stocking, respectively. Finally, within the stocked populations of Orne, Vire, Dordogne, and Garonne, allelic richness ranged from 9 to 13 after stocking.

**Table 2:** Summary per population of population status, stocking intensity (with nonnative fish), main donor stock, allelic richness before, during, and after stocking, effective size (*N*_e_), within river temporal *F*_st_, differentiation among targeted and donor populations, total admixture with other clusters, and persistence of local individuals

Population	Worst population status during last 50 years	Nonnative stocking intensity	Main nonnative donor stock	Allelic richness			*N*_e_		Within river temporal *F*_st_	*F*_st_ with main donor stock^1^			Percentage of admixture with other clusters			Persistence of putative local individuals
			
				Before stocking	Contemporary to stocking	After stocking	Old	Recent		Before stocking	Contemporary to stocking	After stocking	Before stocking	Contemporary to stocking	After stocking	
BRE	Small	High	Scotland	–	10	9	208	64	0.04	–	0.04	0.10	–	60	2	Yes
ARQ	Small	High	Scotland	–	–	8	–	48	–	–	–	0.08	–	–	4	Yes
ORN	Small	Medium	Scotland	–	–	13	–	351	–	–	–	0.03	–	–	62	Yes
VIR	Small	Low	Scotland	–	–	10	–	174	–	–	–	0.03	–	–	50	Yes
SIE	Small	Low	AUL	10	12	–	64	140	0.015	0.06	0.03	–	11	32	–	Yes
SEE	Healthy	Low	AUL	12	12	–	90	189	0.007	0.06	0.05	–	9	14	–	Yes
SEL	Small	Medium	AUL	11	14	–	98	665	0.005	0.06	0.03	–	11	31	–	Yes
COU	Near extinction	High	AUL	8	11	–	73	342	0.023	0.05	0.02	–	18	68	–	Yes
TRI	Small	High	Scotland	–	9	10	271	279	0.034	–	0.04	0.05	–	25	21	Yes
DOU	Small	Medium	Scotland	–	10	11	596	166	0.008	–	0.06	0.04	–	12	11	Yes
ELO	Healthy	Medium	Scotland	–	8	12	393	1289	0.013	–	0.05	0.06	–	11	7	Yes
AUL	Small	High	Scotland	–	9	11	249	402	0.029	–	0.07	0.04	–	18	14	Yes
GOY	Healthy	–	–	–	11	10	225	506	0.001	–	0.05	0.05	–	10	11	Yes
STE	Healthy	Low	Scotland	–	8	9	218	336	0.018	–	0.09	0.04	–	3	8	Yes
JET	Healthy	Low	Scotland	–	6	9	328	685	0.009	–	0.06	0.05	–	4	6	Yes
ODE	Healthy	Low	Scotland	–	8	8	114	612	0.014	–	0.07	0.09	–	3	4	Yes
AVE	Healthy	–	–	–	10	10	165	86	0.011	–	0.06	0.06	–	5	3	Yes
SCO	Healthy	Low	Scotland	–	12	12	188	207	0.007	–	0.06	0.06	–	3	2	Yes
BLA	Healthy	Low	Scotland	–	11	13	585	75	0.002	–	0.07	0.06	–	5	5	Yes
ALL	Small	High	Scotland	–	8	9	409	228	0.016	–	0.10	0.07	–	2	6	Yes
DOR	Near extinction	High	GAV	–	–	9	–	261	–	–	–	0.01	–	–	100	No
GAR	Near extinction	High	ALL	–	–	10	–	81	–	–	–	0.04	–	–	100	No
GAV	Healthy	Medium	Scotland	–	9	12	173	85	0.016	–	0.04	0.04	–	9	11	Yes
NIE	Small	Low	Scotland	–	7	6	126	243	0.001	–	0.08	0.03	–	3	5	Yes
NIL	Small	Medium	Scotland	–	11	8	224	98	0.009	–	0.06	0.02	–	19	17	Yes

1 When the main donor stock was Scottish, the *F*_st_ among SPE and the considered sample was given.

We observed for almost all populations a higher ancestral *θ* than recent *θ* (Table S2), suggesting long-term size reductions in these populations. The recent effective sizes (*N*_e_) were variable among samples (from 48 to 596 for historical samples and from 48 to 1289 for recent ones; Table 2 and Fig. 2). Between the two temporal samples from each population, *N*_e_ decreased in Allier, Bresle, Douron, Aven, and Nive populations and increased in other populations.

### Admixture of populations

**Figure 3:**
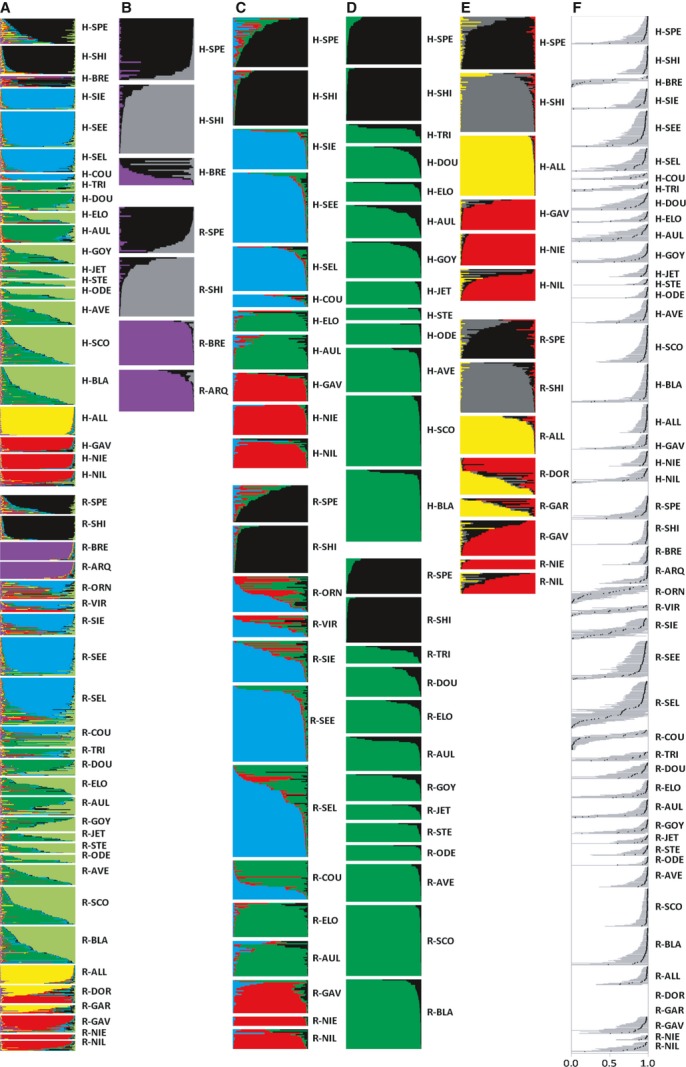
Individual Bayesian clustering results with STRUCTURE for all populations from France for *k* = 7 (A); admixture with donor stocks in Upper-Normandy for *k* = 3 (B); Lower-Normandy for *k* = 4 (C); Brittany for *k* = 2 (D); Allier, Gironde, and Adour for *k* = 4 (E); and admixture values of the local cluster with 90% confidence intervals obtained from B to E analyses (F).

The Bayesian clustering analysis delineated seven genetic groups corresponding to six distinct geographic regions: Scotland, Upper Normandy, Lower Normandy, Brittany, Allier, and Adour (Fig. 3 and Table S3). Individuals from Brittany were grouped into two clusters, suggesting a genetic differentiation between populations located in Northern and Southern coasts of Brittany or a clustering artifact due to the large number of individuals from this geographic region. Admixture rates of stocked populations with each stocking source were then estimated separately for each group of sources of stocking and stocked populations. These admixture rates were highly variable, ranging from 0.01 to 0.61 (Tables 2, S3, and S4). Admixture with the Scottish cluster was 0.60 in the historical sample from the Bresle population at the time of stocking, but only 0.02 in the most recent sample. Admixture rates with the Brittany cluster in the samples from Sienne, Sée, Sélune, and Couesnon, ranged from 0.04 to 0.07 among the historical samples and increased in recently collected samples to 0.18, 0.10, 0.21, and 0.59, respectively. Among populations located in Brittany, admixture with the Scottish cluster ranged from 0.02 to 0.25 and was the highest in samples from Trieux and Aulne rivers. Despite intense stocking, the levels of admixture in historical and contemporary samples from the Allier River with the Scottish and Adour clusters were low (from 0.01 to 0.03). Within samples from the Dordogne and Garonne rivers, admixture rates with the Scottish, Allier and Adour clusters ranged from 0.09 to 0.61 and the analysis did not reveal any additional cluster that may represent the native cluster of both populations. The admixture rates of the Scottish cluster into samples from the Gave D'Oloron, Nive, and Nivelle rivers ranged from 0.02 to 0.13. Finally, 90% credible intervals were relatively small, suggesting a relatively high accuracy of these Bayesian clustering analyses.

### Evolution of the distribution of genetic differentiation within and among populations

**Figure 4:**
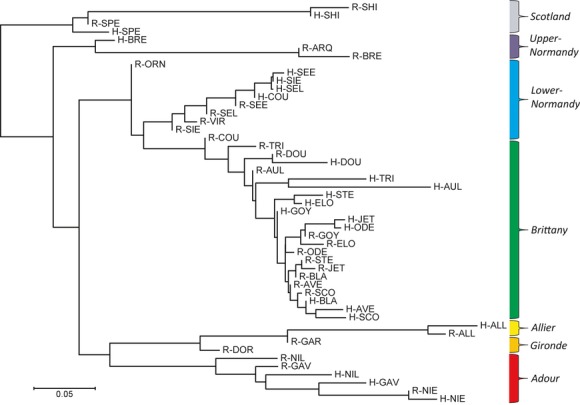
Neighbor-joining tree based on Nei genetic distances among the 49 samples.

The neighbor-joining tree of Nei genetic distances illustrates the genetic structure among French populations (Fig. 4), clearly delineating six groups of French populations (Upper Normandy, Lower Normandy, Brittany, Allier, Gironde, and Adour) plus the Scottish sample, in a similar way as the STRUCTURE analysis. This tree also reveals small genetic distances between the two temporal samples of several populations (e.g., Shin, Spey, Sée, Blavet, Allier, and Nive populations). However, high temporal differentiations were detected in the Bresle, Couesnon, Trieux, and Aulne populations that have been heavily stocked with nonnative fish (Table 2 and Fig. 2). It is especially important to note that the pre-stocking samples from the Couesnon River cluster with other samples from lower Normandy while the post stocking samples cluster with samples from Brittany (Figs. 3 and 4). Similarly, recent samples from Dordogne and Garonne share an intermediate membership from Allier and Adour clusters (Figs. 3 and 4). Accordingly, for several recently stocked populations, the differentiation with donor populations was lower after stocking than before (e.g., Sienne, Sélune, and Couesnon) (Tables 2, S5, and S6). Conversely, the differentiation between recent samples from Bresle and Spey, which were collected several decades after the end of stocking (*F*_st_ = 0.10, CI: 0.09–0.11) was higher than between historical samples of these same rivers, which were collected contemporary to stocking (*F*_st_ = 0.04, CI: 0.03–0.05). The differentiation observed between Bresle and Shine (*F*_st_ = 0.048 and 0.127 for historical and recent samples, respectively) was slightly higher than between Bresle and Soey, but showed a similar increase over time.

**Figure 5:**
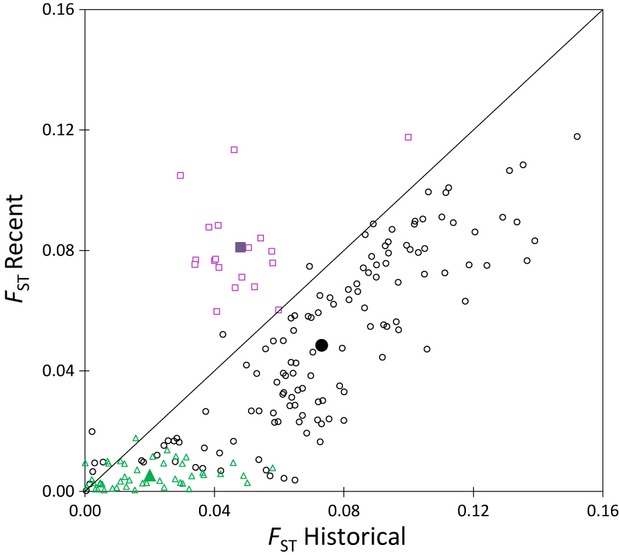
Recent versus historical pairwise genetic differentiation (*F*_st_) among 20 Atlantic salmon populations temporally sampled. The *y* = *x* line indicates the threshold at which *F*_st_ is similar between historical and recent samples. Purple squares indicate pairs including the Bresle population, green triangles indicate pairs including only Brittany populations, and black circles indicate other pairs of populations. Averages for these three groups are given using same but filled symbols.

The genetic differentiation among populations was usually lower in recently collected samples than historical samples in the 20 populations that were sampled temporally (Fig. 5). However, in some pairs, including the Bresle population, we noticed a higher differentiation from all other French populations in recent samples (Fig. 5). Among these 20 populations, we found an overall lower genetic differentiation among recent (*F*_st_ = 0.038, CI: 0.033–0.042) than historical samples (*F*_st_ = 0.063, CI: 0.054–0.071). Analyses of molecular variance furthermore revealed a higher differentiation both among and within groups in historical samples (*F*_CT_ = 0.065, CI: 0.056–0.074 and *F*_SC_ = 0.019, CI: 0. 015–0.023, respectively) than in recent ones (*F*_CT_ = 0.045, CI: 0.039–0.051 and *F*_SC_ = 0.007, CI: 0.006–0009, respectively). In the Brittany region, *F*_st_ among the 13 populations was 0.006, CI: 0.004–0.008 and 0.020, CI: 0.015–0.026 for recent and historical samples, respectively.

## Discussion

This study constitutes one of the largest spatiotemporal assessments of the consequences of stocking practices on the genetic structure of wild populations. In the absence of extensive stocking practices, relatively low gene flow and high stability of the genetic structure among populations or subpopulations of Atlantic salmon have been documented (Vaha et al. 2008; Palstra and Ruzzante 2010). Accordingly, individuals clustered into five geographically distinct groups for both recent and historical periods in the same way as reported in Perrier et al. (2011b) for recent samples. However, in line with Perrier et al. (2011b, 2013) this study confirms that stocking may also have a relatively high impact on the distribution of genetic diversity within and among populations. In particular, we found a lower genetic differentiation among recent samples than among historical samples, which may be linked to stocking, as suggested by previous studies in other species of Salmonids (Eldridge et al. 2009; Pearse et al. 2011; Glover et al. 2012). For example, such reduced differentiation was also observed among Atlantic salmon populations in Norway after gene flow from farmed escapees (Glover et al. 2012). Recent changes in climate may have also affected gene flow among populations, and hence genetic structure, through an increase in dispersal of individuals among populations (Valiente et al. 2010). Also in line with Perrier et al. (2011b, 2013); this study shows variable admixture rates and an increase in genetic diversity in wild populations. Marie et al. (2010) and Lamaze et al. ([Bibr b52][Bibr b53]) studied introgression in Brook trout populations and similarly found an increase in allelic richness with the increase in nonnative stocking intensity in stocked populations. Similar increases in genetic diversity in admixed populations were also observed in other organisms (e.g., the Honey bee, Harpur et al. 2012). Overall, the modifications to genetic structure reported here are in concordance with a number of studies showing that stocking using nonlocal fish can result in variable degrees of modifications in the distribution of genetic diversity within and among populations (Campos et al. 2008; Finnengan and Stevens 2008; Hansen et al. 2009; Marie et al. 2010; Perrier et al. 2011b, 2013).

Although we found high admixture rates in several samples, our results also revealed surprisingly low admixture rates in both historical and recent samples from several heavily stocked rivers including the Allier River and most of the rivers from Brittany. This is in contrast to our predictions as we expected high admixture of both historical and recent samples from the Allier River as a consequence of high stocking intensity of nonlocal fish (mostly from Scotland and Adour) from 1960s to 1990s. A reduced effective population size is expected to further enhance the effects of stocking (Currat et al. 2008; Finnengan and Stevens 2008; Hansen et al. 2009; Marie et al. 2010; Perrier et al. 2013). The size of Allier population has been greatly reduced over the last century, from up to 30,000 fish caught annually on the Loire–Allier system and sold in the 1890s (Bachelier 1963) to approximately 500 fish counted annually on the Allier River in the 2000s. This decrease in census size was congruent with our estimates of historical and contemporary *θ* and of contemporary effective sizes (*N*_e_ = 409, CI: 123–1338 and 228, CI: 72–1216, in the 1960s and the 2000s, respectively). Despite high stocking pressures and a reduced effective population size, we did not detect any significant admixture in either Allier temporal samples. Maladaptation and low fitness of stocked fish have been widely demonstrated and could be one reason for the absence of admixture in this population (McGinnity et al. 1997; Verspoor and de Leaniz 1997; Fleming et al. 2000; Aprahamian et al. 2003; Finnengan and Stevens 2008; Araki et al. 2008). In addition, it has been proposed that the Allier population could represent a special case of local adaptation to a difficult and long migration (major spawning grounds located at 700–900 km from the sea, Perrier et al. 2011b), which may make it especially difficult for stocked fish to reach the spawning grounds and hybridize with native individuals.

Similarly, despite variable supplementation of Brittany populations with fish originating from Scotland from the 1960s to the 1980s, we only detected weak to moderate admixture with the Scottish cluster in most of these populations. Nevertheless, we observed a higher genetic differentiation among populations from Brittany at the time of stocking compared to after its end. A lower genetic differentiation among these populations and Scottish samples was also observed after the end of stocking. While effective population sizes were relatively low (*N*_e_ ≈ 300 on average among old samples), within river *F*_st_s among temporal samples from unstocked rivers were low, thus arguing against the possibility of strong genetic drift. Glover et al. (2012) also established that genetic drift over three decades may only have induced low genetic changes in populations of Atlantic salmon from Norway. We thus propose that stocking using nonnative individuals may have artificially increased the differentiation among populations at first, but that this effect waned when such supplementation practices ended. Natural gene flow among populations may have contributed to the decrease in this inflated structure due to stocking, especially as current global warming may increase salmon straying (Horreo et al. 2011). The potential dispersal of native fish stocked in Aulne and Elorn could also have played a role in decreasing genetic structure given the higher predicted straying of stocked individuals (Quinn 1993; Jonsson et al. 2003). However, the low fitness of stocked fish may also explain the low admixture observed and its potential decrease after the changes of stocking practices.

More markedly, we found a much higher admixture in the historical Bresle sample (60%), collected during stocking, than in the most recent sample (2%), collected several generations after the end of supplementation. Furthermore, we found a higher differentiation between Bresle and Scottish samples (especially Spey) in recently collected samples than in samples collected during stocking. These results strongly suggest that despite high immediate effects of stocking, these practices may only have short-term genetic influences and that a resilience of a “natural” population (neutral) genetic makeup to stocking is possible. While some authors proposed that temporal reproductive isolation (Hendry and Day 2005) may lead to the persistence of native individuals within highly introgressed populations (Hansen and Mensberg 2009), we found some putative hybrids between local and stocked fish in this population. Moreover, the geographic distance among spawning sites is low within this small river and may preclude local isolation by distance among stocked and native fish. Alternatively, the low admixture of the recent sample with the Scottish cluster could be explained by gene flow from nearby populations (Vasemagi et al. 2001; Perrier et al. 2010, 2013). However, this hypothesis seems unlikely given the small size of proximate populations (e.g., Arques) that were also stocked in the same manner. Therefore, the most parsimonious explanation may be that nonlocal stocked fish had lower fitness than wild locally adapted individuals (Araki et al. 2008) and did not contribute much to the contemporary gene pool of the Upper Normandy populations. The decrease in admixture with nonlocal cluster in the contemporary samples from Bresle compared to historical samples, moreover, illustrate that the persistence of locally adapted fish may be an important factor for restoring declining populations.

Our results thus support the hypothesis that stocking can have only low or short-term genetic impacts and that resilience of former patterns of neutral genetic diversity may be possible. Nevertheless, scanning for potential shifts in adaptive genetic variation would be further necessary to investigate the effects of stocking on the evolutionary potential of wild populations (Bourret et al. 2011; Lamaze et al. 2012a,b). Indeed, using genomic cline analyses on a large set of single nucleotide polymorphisms in several stocked Brook trout populations, Lamaze et al. (2012b) detected domestic alleles with either restricted or enhanced introgression rates compared to neutral expectations. These results indicated that selection for several biological processes may have increased or decreased the introgression of domestic genomic regions into wild populations. Different introgression rates depending on loci have also been documented among wild local and translocated domesticated individuals in several other species, notably wolves (Anderson et al. 2009), mouse (Song et al. 2011), and maize (Quist and Chapela 2001). Therefore, the admixture rates described in this study for neutral markers may not be representative of introgression rates at functional genomic regions under different selective pressures between source and stocked populations.

Interspecific hybridization between Atlantic salmon and brown trout has often been documented (Garcia-Vazquez et al. 2003) and may also have occurred in this study system as we were able to detect one hybrid. In fact, stocked fish have been found to participate in hybridization and thus to interspecific introgression (Castillo et al. 2008). Consequently, stocking Atlantic salmon may not only have affected the genetic diversity of this species' populations but also the gene pool of sympatric brown trout populations. Similarly, stocking hatchery-reared brown trout may also have affected genetic diversity within sympatric Atlantic salmon populations. Furthermore, differential introgression has not only been documented among naturally sympatric species (e.g., European oak species, Guichoux et al. 2012) but also among native and introduced species (e.g., introduced and native tiger salamander, Fitzpatrick et al. 2010). Hence, it is probable that stocking one of these two species may change allelic frequencies at some loci conferring higher individual performances. However, several types of incompatibilities, including genetic ones, may exist between Atlantic salmon and Brown trout (Garcia-Vazquez et al. 2004; Álvarez and Garcia-Vazquez 2011), either preventing a high frequency of hybridization events (Maheshwari and Barbash 2011) or generating differential gene flow through the genome. Overall, stocking strategies should take into account the possibility of introgressive hybridization between species.

This study mainly focused on the impacts of nonnative stocking, but the recent supportive-breeding strategy that used hatchery-reared fish from the native river may have also altered the genetic diversity of these populations (Fraser 2008; Araki and Schmid 2010). Indeed, hatchery-reared fish may have a lower genetic diversity than their wild conspecifics, mainly due to the low number of captive breeders relative to wild population sizes, and their release may thus lead to a decrease in the genetic diversity of the stocked population (Verspoor 1988; Wang and Ryman 2001; but see Gow et al. 2011). Such reduced genetic diversity has also been documented in various domesticated species including rabbits (Carneiro et al. 2011), Cattle (Gibbs et al. 2009) and rice (Zhu et al. 2007). However, we did not observe such a decline in genetic diversity in recent samples from Elorn, Allier, and Gave d'Oloron populations that have been recently stocked using native hatchery-reared fish. While we did find a relatively low genetic diversity in recent samples from the Allier population collected at the time of native stocking, the old samples also showed a similarly low diversity. Therefore, we predict that the low genetic diversity in this population is linked to i) its relatively low effective size due to a severe demographic decline and ii) a low immigration rate possibly explained by extensive local adaptation (Perrier et al. 2011b).

This study clearly demonstrates the usefulness of analyzing temporal samples in wild populations to improve their management. We found that stocking nonnative Atlantic salmon was associated with variable, but significant modifications to the distribution of neutral genetic diversity within and among populations at the time of stocking. Although this study did not document the effects of stocking on the functional genetic makeup of wild populations, there is an increasing amount of literature warning on loss of local adaptation and adaptive shifts linked with introgressive hybridization between wild and introduced individuals. It may thus be recommended favoring the use of native and wild individuals (nondomesticated over several generations) to limit detrimental introgressive hybridization. Moreover, neighboring Atlantic salmon populations and even sympatric brown trout populations might also be taken into account given possible intraspecific and interspecific gene flow. That being said, the management programs of already admixed wild populations can also benefit from ameliorations. In agreement with Hansen and Mensberg (2009), this study argues that genetic components of the indigenous population may persist even within highly admixed populations and should be carefully considered as these native individuals may represent the most appropriate basis to restore a locally adapted population. Therefore, if stocking is prolonged to enhance the populations, the remaining native individuals should be identified using genetic analyses and preferred as progenitors in stocking programs. In several contexts of populations enhanced with fish stocked at smolt stage that can be fin clipped, regulations can be undertaken to limit exploitation of wild-born local individuals, and encourage the exploitation of introduced fin-clipped ones. Such regulation is for example accomplished for several Salmonid species in some British Colombia waters (http://www.env.gov.bc.ca/fw/fish/regulations/docs/1113/fishing-synopsis_2011-13.pdf). In the same way, catch and release of Atlantic salmon in healthy populations may also allow conciliating population conservation and game fishing without threatening the wild populations nor requiring compensative stocking (Jensen et al. 2010; Richard et al. 2013). Finally, when habitat quality and connectivity can be restored, the “natural” recolonization of rivers by Atlantic salmon from which it has been extirpated should be favored as a long-term management strategy (Perrier et al. 2010, Griffiths et al. 2011; Ikediashi et al. 2012).

## Supporting Information

**Table S1:** Number of alleles (AN) and observed (Ho) and expected heterozygoties (HE).

**Table S2:** ø.

**Table S3:** *F*st matrix.

**Table S4:** Historical and recent *F*st among target and donor populations that were temporally sampled.

**Table S5:** Genetic clustering of populations using STRUCTURE with k=7.

**Table S6:** Admixture of each sample determined using Structure with separate analyses for estimating admixture in Upper-Normandy, k=3 (I), in Lower-Normandy, k=4 (II), in Brittany, k=2 (III) and in Allier-Gironde-Adour, k=4 (IV). Local clusters given in bold, stocked samples highlighted in grey.
