# Supplementary material for: Changes in the genetic structure of Atlantic salmon populations over four decades reveal substantial impacts of stocking and potential resiliency
Source: Ecol Evol. 2013 Jun 12;3(7):2334–49. doi: 10.1002/ece3.629 (PMC3728969; doi:10.1002/ece3.629)
Supplement: Supplementary file 1 [file ece30003-2334-SD1.docx]

Table S1: Number of alleles (AN) and observed (Ho) and expected heterozygoties (HE). Significant inbreeding coefficient (FIS) are given in bold, and in italic if possibly associated with null alleles.

|  |  | SSA0021NVH | SSA0071NVH | SSA85 | SSOSL85 | SSA0217NVH | SSA0008NVH | SSA197 | SSA171 | SSA0057NVH | SSSP2216 | SSA224 | Average |
| --- | --- | --- | --- | --- | --- | --- | --- | --- | --- | --- | --- | --- | --- |
| BRE68 | AN | 5 | 10 | 11 | 11 | 14 | 8 | 15 | 8 | 16 | 11 | 6 | 10 |
|  | Ho | 0.68 | 0.79 | 0.83 | 0.63 | 0.95 | 0.63 | 0.63 | 0.75 | 0.95 | 0.74 | 0.56 | 0.74 |
|  | He | 0.79 | 0.84 | 0.91 | 0.83 | 0.92 | 0.74 | 0.93 | 0.85 | 0.93 | 0.87 | 0.7 | 0.85 |
|  | Fis | 0.13 | 0.07 | 0.09 | *0.25* | -0.03 | 0.15 | ***0.33*** | 0.12 | -0.02 | 0.16 | 0.21 | 0.13 |
| BRE03 | AN | 6 | 11 | 9 | 8 | 13 | 5 | 10 | 11 | 14 | 9 | 6 | 9 |
|  | Ho | 0.87 | 0.83 | 0.9 | 0.83 | 0.86 | 0.5 | 0.69 | 0.87 | 0.66 | 0.76 | 0.7 | 0.77 |
|  | He | 0.78 | 0.83 | 0.8 | 0.8 | 0.85 | 0.52 | 0.73 | 0.85 | 0.87 | 0.77 | 0.74 | 0.78 |
|  | Fis | -0.11 | 0 | -0.12 | -0.04 | -0.02 | 0.05 | 0.05 | -0.02 | *0.25* | 0.02 | 0.06 | 0.01 |
| ARQ03 | AN | 4 | 9 | 8 | 7 | 12 | 5 | 9 | 8 | 10 | 8 | 7 | 8 |
|  | Ho | 0.68 | 0.81 | 0.57 | 0.84 | 0.84 | 0.53 | 0.68 | 0.73 | 0.71 | 0.8 | 0.84 | 0.73 |
|  | He | 0.68 | 0.85 | 0.7 | 0.83 | 0.85 | 0.45 | 0.74 | 0.81 | 0.84 | 0.83 | 0.76 | 0.76 |
|  | Fis | 0 | 0.06 | 0.19 | -0.02 | 0.02 | **-0.2** | 0.08 | 0.1 | 0.16 | 0.03 | -0.1 | 0.03 |
| ORN03 | AN | 5 | 14 | 18 | 13 | 19 | 6 | 12 | 12 | 21 | 12 | 6 | 13 |
|  | Ho | 0.87 | 0.84 | 0.93 | 0.97 | 0.87 | 0.74 | 0.81 | 0.9 | 0.73 | 0.8 | 0.7 | 0.83 |
|  | He | 0.75 | 0.91 | 0.92 | 0.89 | 0.94 | 0.75 | 0.83 | 0.88 | 0.94 | 0.89 | 0.68 | 0.85 |
|  | Fis | -0.16 | 0.08 | -0.02 | -0.09 | 0.07 | 0.01 | 0.03 | -0.03 | ***0.23*** | 0.11 | -0.03 | 0.02 |
| VIR03 | AN | 4 | 10 | 12 | 10 | 15 | 8 | 13 | 11 | 15 | 11 | 4 | 10 |
|  | Ho | 0.58 | 0.79 | 0.79 | 0.84 | 0.95 | 0.84 | 0.79 | 0.88 | 0.89 | 0.89 | 0.63 | 0.81 |
|  | He | 0.74 | 0.86 | 0.9 | 0.88 | 0.92 | 0.84 | 0.88 | 0.9 | 0.92 | 0.87 | 0.59 | 0.85 |
|  | Fis | 0.22 | 0.09 | 0.12 | 0.05 | -0.04 | -0.01 | 0.1 | 0.02 | 0.03 | -0.03 | -0.07 | 0.04 |
| SIE86 | AN | 3 | 11 | 11 | 14 | 16 | 9 | 7 | 9 | 15 | 11 | 5 | 10 |
|  | Ho | 0.57 | 0.85 | 0.77 | 0.9 | 0.97 | 0.8 | 0.8 | 0.76 | 0.8 | 0.88 | 0.47 | 0.78 |
|  | He | 0.47 | 0.83 | 0.86 | 0.88 | 0.89 | 0.77 | 0.79 | 0.81 | 0.87 | 0.79 | 0.52 | 0.77 |
|  | Fis | -0.23 | -0.03 | 0.1 | -0.03 | -0.1 | -0.03 | -0.02 | 0.06 | 0.08 | -0.12 | 0.1 | -0.02 |
| SIE03 | AN | 5 | 12 | 12 | 13 | 17 | 7 | 13 | 15 | 22 | 12 | 7 | 12 |
|  | Ho | 0.68 | 0.86 | 0.86 | 0.92 | 0.92 | 0.54 | 0.81 | 0.89 | 1 | 0.86 | 0.72 | 0.82 |
|  | He | 0.68 | 0.86 | 0.83 | 0.87 | 0.9 | 0.68 | 0.83 | 0.88 | 0.95 | 0.86 | 0.64 | 0.82 |
|  | Fis | 0 | 0 | -0.04 | -0.05 | -0.01 | *0.21* | 0.03 | -0.01 | **-0.06** | -0.01 | -0.13 | -0.01 |
| SEE77 | AN | 5 | 10 | 12 | 16 | 17 | 8 | 9 | 12 | 20 | 16 | 7 | 12 |
|  | Ho | 0.44 | 0.84 | 0.78 | 0.73 | 0.93 | 0.75 | 0.8 | 0.67 | 0.97 | 0.72 | 0.65 | 0.75 |
|  | He | 0.49 | 0.82 | 0.86 | 0.86 | 0.9 | 0.76 | 0.75 | 0.82 | 0.92 | 0.86 | 0.59 | 0.78 |
|  | Fis | 0.1 | -0.02 | 0.08 | *0.16* | -0.04 | 0.01 | -0.08 | *0.19* | -0.06 | *0.16* | -0.1 | 0.04 |
| SEE03 | AN | 4 | 12 | 17 | 13 | 19 | 8 | 8 | 11 | 19 | 13 | 5 | 12 |
|  | Ho | 0.57 | 0.73 | 0.79 | 0.85 | 0.89 | 0.79 | 0.88 | 0.8 | 0.88 | 0.84 | 0.63 | 0.79 |
|  | He | 0.63 | 0.83 | 0.86 | 0.88 | 0.89 | 0.71 | 0.78 | 0.8 | 0.9 | 0.79 | 0.62 | 0.79 |
|  | Fis | 0.1 | *0.12* | 0.08 | 0.03 | 0 | -0.11 | -0.12 | 0.01 | 0.02 | -0.06 | -0.03 | 0.00 |
| SEL77 | AN | 5 | 11 | 12 | 12 | 19 | 8 | 8 | 6 | 18 | 15 | 5 | 11 |
|  | Ho | 0.51 | 0.69 | 0.82 | 0.79 | 0.92 | 0.69 | 0.77 | 0.74 | 0.82 | 0.68 | 0.49 | 0.72 |
|  | He | 0.6 | 0.86 | 0.87 | 0.87 | 0.92 | 0.69 | 0.8 | 0.79 | 0.91 | 0.82 | 0.5 | 0.78 |
|  | Fis | 0.15 | *0.2* | 0.07 | 0.1 | 0 | 0 | 0.03 | 0.06 | 0.1 | *0.17* | 0.02 | 0.08 |
| SEL03 | AN | 4 | 15 | 17 | 17 | 20 | 9 | 15 | 14 | 22 | 13 | 8 | 14 |
|  | Ho | 0.69 | 0.84 | 0.85 | 0.89 | 0.9 | 0.76 | 0.79 | 0.79 | 0.95 | 0.87 | 0.72 | 0.82 |
|  | He | 0.64 | 0.9 | 0.86 | 0.89 | 0.92 | 0.71 | 0.82 | 0.83 | 0.92 | 0.84 | 0.66 | 0.82 |
|  | Fis | -0.07 | 0.07 | 0.01 | -0.01 | 0.02 | -0.07 | 0.04 | 0.05 | -0.03 | -0.04 | -0.1 | -0.01 |
| COU82 | AN | 3 | 7 | 8 | 10 | 12 | 6 | 6 | 8 | 10 | 12 | 6 | 8 |
|  | Ho | 0.64 | 0.88 | 0.75 | 0.82 | 0.91 | 0.45 | 0.73 | 0.91 | 1 | 0.73 | 0.73 | 0.78 |
|  | He | 0.61 | 0.88 | 0.88 | 0.9 | 0.94 | 0.63 | 0.8 | 0.87 | 0.9 | 0.83 | 0.71 | 0.81 |
|  | Fis | -0.05 | 0.01 | 0.16 | 0.1 | 0.04 | 0.29 | 0.1 | -0.05 | **-0.11** | 0.13 | -0.03 | 0.05 |
| COU03 | AN | 4 | 12 | 13 | 15 | 14 | 7 | 11 | 11 | 18 | 12 | 6 | 11 |
|  | Ho | 0.71 | 0.82 | 0.88 | 0.87 | 0.88 | 0.74 | 0.76 | 0.77 | 0.91 | 0.88 | 0.82 | 0.82 |
|  | He | 0.7 | 0.88 | 0.85 | 0.9 | 0.88 | 0.71 | 0.82 | 0.8 | 0.92 | 0.87 | 0.68 | 0.82 |
|  | Fis | -0.01 | 0.07 | -0.04 | 0.03 | -0.01 | -0.03 | 0.07 | 0.04 | 0.01 | -0.02 | -0.22 | -0.01 |
| TRI77 | AN | 5 | 8 | 12 | 8 | 12 | 6 | 10 | 9 | 13 | 9 | 6 | 9 |
|  | Ho | 0.47 | 0.9 | 0.82 | 0.76 | 0.94 | 0.76 | 0.82 | 0.73 | 0.82 | 0.81 | 0.64 | 0.77 |
|  | He | 0.67 | 0.75 | 0.86 | 0.84 | 0.87 | 0.73 | 0.84 | 0.84 | 0.89 | 0.88 | 0.71 | 0.81 |
|  | Fis | 0.3 | -0.22 | 0.04 | 0.1 | -0.09 | -0.04 | 0.02 | 0.14 | 0.07 | 0.08 | 0.09 | 0.04 |
| TRI03 | AN | 4 | 11 | 13 | 11 | 16 | 6 | 12 | 11 | 12 | 11 | 5 | 10 |
|  | Ho | 0.64 | 0.81 | 0.96 | 0.73 | 0.88 | 0.72 | 0.85 | 0.94 | 0.94 | 0.88 | 0.69 | 0.82 |
|  | He | 0.68 | 0.83 | 0.87 | 0.86 | 0.91 | 0.78 | 0.87 | 0.9 | 0.89 | 0.91 | 0.7 | 0.84 |
|  | Fis | 0.05 | 0.03 | -0.11 | 0.16 | 0.03 | 0.08 | 0.02 | -0.05 | -0.06 | 0.04 | 0.02 | 0.02 |
| DOU82 | AN | 5 | 8 | 11 | 10 | 13 | 6 | 10 | 10 | 18 | 11 | 6 | 10 |
|  | Ho | 0.45 | 0.78 | 0.68 | 0.86 | 0.89 | 0.79 | 0.86 | 0.74 | 0.79 | 0.86 | 0.64 | 0.76 |
|  | He | 0.7 | 0.86 | 0.8 | 0.88 | 0.91 | 0.7 | 0.83 | 0.82 | 0.92 | 0.87 | 0.61 | 0.81 |
|  | Fis | *0.36* | 0.1 | 0.15 | 0.02 | 0.01 | -0.13 | -0.04 | 0.1 | *0.14* | 0.01 | -0.05 | 0.06 |
| DOU03 | AN | 5 | 15 | 12 | 13 | 15 | 6 | 13 | 10 | 14 | 13 | 5 | 11 |
|  | Ho | 0.78 | 0.89 | 0.88 | 0.96 | 0.85 | 0.67 | 0.85 | 0.96 | 0.81 | 0.78 | 0.7 | 0.83 |
|  | He | 0.71 | 0.93 | 0.88 | 0.89 | 0.93 | 0.7 | 0.86 | 0.87 | 0.88 | 0.89 | 0.64 | 0.83 |
|  | Fis | -0.11 | 0.04 | 0 | -0.09 | 0.08 | 0.05 | 0.01 | -0.12 | 0.08 | 0.13 | -0.1 | 0.00 |
| ELO75 | AN | 4 | 4 | 9 | 10 | 9 | 6 | 12 | 7 | 13 | 10 | 6 | 8 |
|  | Ho | 0.7 | 0.56 | 0.72 | 0.6 | 0.9 | 0.7 | 0.83 | 0.73 | 0.83 | 0.67 | 0.8 | 0.73 |
|  | He | 0.66 | 0.7 | 0.81 | 0.69 | 0.8 | 0.69 | 0.84 | 0.85 | 0.86 | 0.84 | 0.63 | 0.76 |
|  | Fis | -0.06 | 0.22 | 0.11 | 0.13 | -0.13 | -0.01 | 0 | 0.14 | 0.03 | *0.21* | -0.28 | 0.03 |
| ELO03 | AN | 4 | 13 | 14 | 13 | 17 | 6 | 12 | 15 | 20 | 11 | 4 | 12 |
|  | Ho | 0.87 | 0.73 | 0.93 | 0.79 | 0.94 | 0.74 | 0.85 | 0.78 | 0.79 | 0.88 | 0.76 | 0.82 |
|  | He | 0.73 | 0.84 | 0.88 | 0.83 | 0.92 | 0.74 | 0.82 | 0.89 | 0.93 | 0.87 | 0.66 | 0.83 |
|  | Fis | -0.2 | 0.13 | -0.07 | 0.05 | -0.03 | 0 | -0.04 | 0.12 | *0.15* | -0.01 | -0.15 | 0.00 |
| AUL69 | AN | 5 | 5 | 9 | 7 | 15 | 6 | 10 | 9 | 14 | 10 | 4 | 9 |
|  | Ho | 0.83 | 0.83 | 0.72 | 0.78 | 0.94 | 0.78 | 0.67 | 0.73 | 0.83 | 0.78 | 0.67 | 0.78 |
|  | He | 0.71 | 0.79 | 0.8 | 0.81 | 0.92 | 0.74 | 0.81 | 0.88 | 0.92 | 0.84 | 0.61 | 0.80 |
|  | Fis | -0.18 | -0.06 | 0.1 | 0.04 | -0.03 | -0.06 | 0.19 | 0.18 | 0.1 | 0.08 | -0.09 | 0.02 |
| AUL03 | AN | 4 | 11 | 13 | 12 | 13 | 7 | 11 | 14 | 18 | 12 | 4 | 11 |
|  | Ho | 0.59 | 0.79 | 0.94 | 0.82 | 0.73 | 0.79 | 0.73 | 0.9 | 0.87 | 0.8 | 0.6 | 0.78 |
|  | He | 0.67 | 0.8 | 0.9 | 0.85 | 0.82 | 0.77 | 0.8 | 0.91 | 0.85 | 0.85 | 0.67 | 0.81 |
|  | Fis | 0.12 | 0.01 | -0.05 | 0.04 | 0.12 | -0.02 | 0.09 | 0.01 | -0.02 | 0.06 | 0.1 | 0.04 |
| GOY81 | AN | 5 | 12 | 10 | 11 | 16 | 7 | 13 | 9 | 21 | 14 | 4 | 11 |
|  | Ho | 0.67 | 0.84 | 0.76 | 0.88 | 0.85 | 0.76 | 0.88 | 0.9 | 0.81 | 0.85 | 0.85 | 0.82 |
|  | He | 0.75 | 0.87 | 0.87 | 0.85 | 0.9 | 0.7 | 0.81 | 0.86 | 0.89 | 0.86 | 0.69 | 0.82 |
|  | Fis | 0.11 | 0.04 | 0.13 | -0.03 | 0.06 | -0.09 | -0.08 | -0.05 | 0.09 | 0.01 | -0.23 | 0.00 |
| GOY03 | AN | 4 | 12 | 11 | 11 | 16 | 5 | 12 | 11 | 14 | 12 | 4 | 10 |
|  | Ho | 0.53 | 0.85 | 0.85 | 0.9 | 0.76 | 0.62 | 0.94 | 0.83 | 0.83 | 0.75 | 0.67 | 0.78 |
|  | He | 0.65 | 0.85 | 0.83 | 0.87 | 0.88 | 0.65 | 0.87 | 0.88 | 0.91 | 0.85 | 0.65 | 0.81 |
|  | Fis | 0.19 | -0.01 | -0.02 | -0.04 | 0.13 | 0.06 | -0.09 | 0.05 | 0.09 | 0.12 | -0.03 | 0.04 |
| STE72 | AN | 5 | 7 | 7 | 12 | 12 | 5 | 10 | 4 | 14 | 10 | 6 | 8 |
|  | Ho | 0.62 | 0.6 | 0.71 | 0.81 | 0.95 | 0.48 | 0.86 | 0.83 | 0.9 | 0.67 | 0.67 | 0.74 |
|  | He | 0.62 | 0.78 | 0.76 | 0.89 | 0.89 | 0.58 | 0.84 | 0.71 | 0.89 | 0.88 | 0.6 | 0.77 |
|  | Fis | 0 | 0.23 | 0.06 | 0.09 | -0.08 | 0.19 | -0.03 | -0.19 | -0.01 | *0.25* | -0.12 | 0.04 |
| STE03 | AN | 5 | 10 | 9 | 10 | 11 | 4 | 12 | 9 | 13 | 9 | 6 | 9 |
|  | Ho | 0.6 | 0.85 | 0.84 | 1 | 0.84 | 0.65 | 0.9 | 0.83 | 0.92 | 0.92 | 0.86 | 0.84 |
|  | He | 0.68 | 0.89 | 0.84 | 0.86 | 0.88 | 0.71 | 0.86 | 0.87 | 0.93 | 0.89 | 0.75 | 0.83 |
|  | Fis | 0.12 | 0.05 | 0 | **-0.17** | 0.05 | 0.08 | -0.05 | 0.04 | 0.01 | -0.04 | -0.16 | -0.01 |
| JET72 | AN | 4 | 6 | 5 | 9 | 9 | 5 | 8 | 4 | 8 | 8 | 4 | 6 |
|  | Ho | 0.91 | 0.78 | 0.91 | 0.67 | 0.82 | 0.73 | 0.91 | 0.67 | 1 | 0.56 | 0.45 | 0.76 |
|  | He | 0.73 | 0.8 | 0.77 | 0.89 | 0.85 | 0.74 | 0.84 | 0.8 | 0.79 | 0.88 | 0.57 | 0.79 |
|  | Fis | -0.26 | 0.03 | -0.2 | 0.26 | 0.04 | 0.01 | -0.08 | **0.2** | **-0.28** | 0.38 | 0.21 | 0.03 |
| JET03 | AN | 4 | 9 | 9 | 9 | 14 | 4 | 10 | 9 | 14 | 9 | 4 | 9 |
|  | Ho | 0.6 | 0.8 | 0.92 | 0.7 | 0.9 | 0.6 | 0.75 | 0.76 | 1 | 1 | 0.76 | 0.80 |
|  | He | 0.67 | 0.83 | 0.83 | 0.86 | 0.9 | 0.63 | 0.78 | 0.8 | 0.89 | 0.87 | 0.63 | 0.79 |
|  | Fis | 0.11 | 0.04 | -0.12 | 0.19 | 0 | 0.05 | 0.04 | 0.04 | **-0.13** | **-0.15** | -0.22 | -0.01 |
| ODE72 | AN | 4 | 3 | 7 | 9 | 10 | 6 | 12 | 6 | 16 | 12 | 6 | 8 |
|  | Ho | 0.79 | 0.8 | 0.74 | 0.74 | 0.89 | 0.84 | 0.74 | 0.73 | 1 | 0.58 | 0.74 | 0.78 |
|  | He | 0.67 | 0.62 | 0.78 | 0.88 | 0.84 | 0.72 | 0.82 | 0.76 | 0.91 | 0.83 | 0.67 | 0.77 |
|  | Fis | -0.19 | **-0.33** | 0.05 | 0.17 | -0.07 | -0.18 | 0.1 | 0.05 | **-0.1** | *0.31* | -0.11 | -0.03 |
| ODE03 | AN | 5 | 11 | 6 | 11 | 11 | 5 | 10 | 11 | 10 | 10 | 3 | 8 |
|  | Ho | 0.53 | 0.89 | 0.68 | 0.89 | 0.94 | 0.67 | 0.79 | 0.71 | 0.93 | 0.79 | 0.71 | 0.78 |
|  | He | 0.7 | 0.88 | 0.78 | 0.86 | 0.89 | 0.62 | 0.77 | 0.91 | 0.85 | 0.88 | 0.54 | 0.79 |
|  | Fis | 0.26 | -0.02 | 0.13 | -0.04 | -0.06 | -0.08 | -0.03 | *0.22* | -0.1 | 0.11 | -0.33 | 0.01 |
| AVE77 | AN | 4 | 8 | 11 | 10 | 14 | 6 | 11 | 11 | 16 | 10 | 4 | 10 |
|  | Ho | 0.7 | 0.85 | 0.78 | 0.73 | 0.83 | 0.7 | 0.75 | 0.86 | 0.88 | 0.8 | 0.75 | 0.78 |
|  | He | 0.63 | 0.86 | 0.83 | 0.78 | 0.83 | 0.68 | 0.81 | 0.84 | 0.92 | 0.85 | 0.74 | 0.80 |
|  | Fis | -0.11 | 0.01 | 0.06 | 0.07 | 0.01 | -0.03 | 0.07 | -0.03 | 0.05 | 0.06 | -0.02 | 0.01 |
| AVE03 | AN | 4 | 12 | 11 | 11 | 15 | 7 | 9 | 13 | 17 | 12 | 4 | 10 |
|  | Ho | 0.65 | 0.91 | 0.76 | 0.79 | 0.85 | 0.65 | 0.82 | 0.79 | 0.76 | 0.97 | 0.82 | 0.80 |
|  | He | 0.72 | 0.88 | 0.82 | 0.84 | 0.87 | 0.69 | 0.77 | 0.86 | 0.9 | 0.87 | 0.67 | 0.81 |
|  | Fis | 0.1 | -0.04 | 0.07 | 0.05 | 0.02 | 0.07 | -0.08 | 0.08 | *0.15* | -0.12 | -0.24 | 0.01 |
| SCO77 | AN | 5 | 12 | 13 | 11 | 15 | 6 | 15 | 12 | 19 | 16 | 4 | 12 |
|  | Ho | 0.7 | 0.8 | 0.9 | 0.71 | 0.7 | 0.63 | 0.77 | 0.63 | 0.86 | 0.84 | 0.64 | 0.74 |
|  | He | 0.71 | 0.88 | 0.84 | 0.8 | 0.76 | 0.65 | 0.8 | 0.79 | 0.92 | 0.87 | 0.66 | 0.79 |
|  | Fis | 0.02 | 0.08 | -0.08 | 0.12 | 0.07 | 0.04 | 0.05 | *0.2* | 0.07 | 0.03 | 0.04 | 0.06 |
| SCO03 | AN | 4 | 14 | 12 | 15 | 15 | 6 | 16 | 13 | 19 | 14 | 5 | 12 |
|  | Ho | 0.62 | 0.88 | 0.8 | 0.88 | 0.76 | 0.7 | 0.86 | 0.87 | 0.86 | 0.83 | 0.67 | 0.79 |
|  | He | 0.66 | 0.89 | 0.84 | 0.86 | 0.84 | 0.68 | 0.84 | 0.85 | 0.9 | 0.86 | 0.66 | 0.81 |
|  | Fis | 0.06 | 0.01 | 0.05 | -0.03 | 0.09 | -0.03 | -0.02 | -0.02 | 0.04 | 0.03 | -0.02 | 0.01 |
| BLA77 | AN | 4 | 11 | 9 | 11 | 16 | 7 | 13 | 13 | 20 | 13 | 5 | 11 |
|  | Ho | 0.69 | 0.83 | 0.8 | 0.7 | 0.8 | 0.6 | 0.88 | 0.7 | 0.92 | 0.89 | 0.6 | 0.76 |
|  | He | 0.7 | 0.84 | 0.82 | 0.84 | 0.87 | 0.64 | 0.83 | 0.81 | 0.92 | 0.86 | 0.63 | 0.80 |
|  | Fis | 0.01 | 0.02 | 0.02 | *0.17* | 0.08 | 0.06 | -0.06 | *0.14* | -0.01 | -0.04 | 0.04 | 0.04 |
| BLA03 | AN | 5 | 13 | 15 | 13 | 17 | 7 | 17 | 13 | 21 | 15 | 5 | 13 |
|  | Ho | 0.68 | 0.81 | 0.85 | 0.88 | 0.87 | 0.59 | 0.92 | 0.82 | 0.86 | 0.83 | 0.59 | 0.79 |
|  | He | 0.68 | 0.87 | 0.84 | 0.85 | 0.88 | 0.65 | 0.86 | 0.85 | 0.9 | 0.88 | 0.66 | 0.81 |
|  | Fis | 0 | 0.07 | -0.02 | -0.03 | 0.01 | 0.1 | -0.07 | 0.04 | 0.05 | 0.06 | 0.1 | 0.03 |
| ALL67 | AN | 5 | 8 | 6 | 9 | 13 | 5 | 10 | 8 | 13 | 7 | 5 | 8 |
|  | Ho | 0.84 | 0.7 | 0.71 | 0.86 | 0.82 | 0.71 | 0.84 | 0.62 | 0.9 | 0.61 | 0.63 | 0.75 |
|  | He | 0.68 | 0.76 | 0.6 | 0.79 | 0.88 | 0.76 | 0.79 | 0.76 | 0.89 | 0.75 | 0.57 | 0.75 |
|  | Fis | -0.24 | 0.07 | -0.19 | -0.08 | 0.08 | 0.06 | -0.06 | *0.19* | 0 | *0.19* | -0.1 | -0.01 |
| ALL03 | AN | 5 | 8 | 9 | 8 | 11 | 7 | 11 | 9 | 13 | 9 | 5 | 9 |
|  | Ho | 0.62 | 0.83 | 0.85 | 0.83 | 0.89 | 0.6 | 0.74 | 0.71 | 0.81 | 0.74 | 0.84 | 0.77 |
|  | He | 0.71 | 0.81 | 0.78 | 0.76 | 0.85 | 0.68 | 0.76 | 0.81 | 0.85 | 0.78 | 0.74 | 0.78 |
|  | Fis | 0.13 | -0.03 | -0.1 | -0.09 | -0.05 | 0.12 | 0.02 | 0.12 | 0.06 | 0.05 | -0.14 | 0.01 |
| DOR03 | AN | 4 | 9 | 9 | 11 | 10 | 6 | 11 | 10 | 13 | 7 | 4 | 9 |
|  | Ho | 0.8 | 0.87 | 0.73 | 0.93 | 0.93 | 0.67 | 0.87 | 1 | 1 | 0.92 | 0.83 | 0.87 |
|  | He | 0.77 | 0.86 | 0.73 | 0.91 | 0.88 | 0.63 | 0.9 | 0.87 | 0.92 | 0.86 | 0.73 | 0.82 |
|  | Fis | -0.04 | 0 | 0 | -0.03 | -0.07 | -0.06 | 0.04 | **-0.16** | **-0.1** | -0.08 | -0.15 | -0.06 |
| GAR03 | AN | 4 | 10 | 13 | 11 | 14 | 10 | 16 | 8 | 13 | 10 | 5 | 10 |
|  | Ho | 0.8 | 0.82 | 0.83 | 0.9 | 0.93 | 0.72 | 0.93 | 0.81 | 0.81 | 0.9 | 0.81 | 0.84 |
|  | He | 0.76 | 0.87 | 0.81 | 0.84 | 0.91 | 0.77 | 0.91 | 0.81 | 0.83 | 0.86 | 0.59 | 0.81 |
|  | Fis | -0.06 | 0.05 | -0.03 | -0.07 | -0.02 | 0.06 | -0.03 | 0 | 0.03 | -0.05 | **-0.37** | -0.04 |
| GAV84 | AN | 4 | 9 | 9 | 7 | 13 | 6 | 10 | 10 | 14 | 12 | 6 | 9 |
|  | Ho | 0.72 | 0.84 | 0.92 | 0.68 | 0.68 | 0.76 | 0.88 | 0.75 | 0.76 | 0.92 | 0.8 | 0.79 |
|  | He | 0.68 | 0.81 | 0.88 | 0.79 | 0.87 | 0.79 | 0.88 | 0.8 | 0.88 | 0.88 | 0.72 | 0.82 |
|  | Fis | -0.06 | -0.03 | -0.05 | 0.14 | *0.23* | 0.04 | 0 | 0.07 | 0.13 | -0.04 | -0.12 | 0.03 |
| GAV03 | AN | 4 | 10 | 13 | 10 | 16 | 6 | 15 | 13 | 21 | 15 | 6 | 12 |
|  | Ho | 0.83 | 0.9 | 0.86 | 0.72 | 0.83 | 0.59 | 0.93 | 0.93 | 0.79 | 0.93 | 0.62 | 0.81 |
|  | He | 0.68 | 0.87 | 0.89 | 0.83 | 0.92 | 0.78 | 0.9 | 0.93 | 0.91 | 0.91 | 0.6 | 0.84 |
|  | Fis | -0.23 | -0.03 | 0.03 | 0.14 | 0.1 | *0.25* | -0.03 | -0.01 | 0.13 | -0.02 | -0.03 | 0.03 |
| NIE84 | AN | 4 | 7 | 8 | 7 | 10 | 6 | 9 | 8 | 7 | 9 | 4 | 7 |
|  | Ho | 0.69 | 0.83 | 0.88 | 0.81 | 0.85 | 0.73 | 0.92 | 0.63 | 0.79 | 0.8 | 0.42 | 0.76 |
|  | He | 0.64 | 0.73 | 0.86 | 0.81 | 0.84 | 0.72 | 0.83 | 0.66 | 0.75 | 0.83 | 0.47 | 0.74 |
|  | Fis | -0.09 | -0.15 | -0.03 | 0.01 | -0.01 | -0.01 | -0.12 | 0.05 | -0.06 | 0.03 | 0.09 | -0.03 |
| NIE03 | AN | 4 | 6 | 6 | 7 | 8 | 6 | 7 | 5 | 5 | 7 | 3 | 6 |
|  | Ho | 0.88 | 0.75 | 0.88 | 0.88 | 0.88 | 0.5 | 0.88 | 0.71 | 0.86 | 1 | 0.29 | 0.77 |
|  | He | 0.69 | 0.74 | 0.85 | 0.89 | 0.9 | 0.82 | 0.88 | 0.81 | 0.7 | 0.85 | 0.48 | 0.78 |
|  | Fis | -0.29 | -0.01 | -0.03 | 0.02 | 0.03 | 0.4 | 0 | 0.13 | -0.24 | **-0.2** | 0.43 | 0.02 |
| NIL80 | AN | 4 | 8 | 12 | 14 | 16 | 7 | 11 | 11 | 18 | 13 | 6 | 11 |
|  | Ho | 0.65 | 0.95 | 0.77 | 0.76 | 0.88 | 0.81 | 0.73 | 0.72 | 0.81 | 0.76 | 0.56 | 0.76 |
|  | He | 0.68 | 0.86 | 0.85 | 0.86 | 0.85 | 0.79 | 0.87 | 0.81 | 0.9 | 0.9 | 0.6 | 0.82 |
|  | Fis | 0.04 | -0.12 | 0.09 | 0.12 | -0.04 | -0.02 | 0.16 | 0.11 | 0.1 | *0.16* | 0.07 | 0.06 |
| NIL03 | AN | 5 | 10 | 8 | 6 | 9 | 4 | 12 | 8 | 14 | 9 | 4 | 8 |
|  | Ho | 0.59 | 0.88 | 0.76 | 0.76 | 0.88 | 0.88 | 0.94 | 0.92 | 0.69 | 0.92 | 0.92 | 0.83 |
|  | He | 0.73 | 0.9 | 0.87 | 0.71 | 0.81 | 0.75 | 0.89 | 0.85 | 0.93 | 0.88 | 0.74 | 0.82 |
|  | Fis | 0.2 | 0.02 | 0.12 | -0.08 | -0.08 | -0.18 | -0.06 | -0.09 | *0.27* | -0.06 | -0.26 | -0.02 |

Table S2. θ.

|  |  | | | |
| --- | --- | --- | --- | --- |
|  | Ancestral θ | | Recent θ | |
|  | Historical sample Recent sample | | Historical sample Recent sample | |
| BRE | 89 | 64 | 21 | 9 |
| ARQ | - | 59 | - | 8 |
| ORN | - | 97 | - | 23 |
| VIR | - | 101 | - | 20 |
| SIE | 91 | 117 | 9 | 14 |
| SEE | 101 | 97 | 10 | 11 |
| SEL | 106 | 105 | 11 | 15 |
| COU | 105 | 104 | 14 | 15 |
| TRI | 80 | 82 | 13 | 17 |
| DOU | 90 | 100 | 13 | 18 |
| ELO | 73 | 81 | 12 | 13 |
| AUL | 75 | 86 | 8 | 16 |
| GOY | 77 | 81 | 16 | 14 |
| STE | 79 | 82 | 8 | 17 |
| JET | 53 | 58 | 10 | 12 |
| ODE | 85 | 69 | 9 | 10 |
| AVE | 52 | 75 | 12 | 13 |
| SCO | 83 | 99 | 11 | 21 |
| BLA | 80 | 97 | 11 | 14 |
| ALL | 62 | 58 | 7 | 10 |
| DOR | - | 65 | - | 16 |
| GAR | - | 81 | - | 14 |
| GAV | 78 | 104 | 14 | 19 |
| NIE | 57 | 67 | 7 | 10 |
| NIL | 98 | 71 | 14 | 15 |

Table S3. *F*_ST_ matrix.

|  | HSPE | HSHI | HBRE | HSIE | HSEE | HSEL | HCOU | HTRI | HDOU | HELO | HAUL | HGOY | HJET | HSTE | HODE | HAVE | HSCO | HBLA | HALL | HGAV | HNIE |
| --- | --- | --- | --- | --- | --- | --- | --- | --- | --- | --- | --- | --- | --- | --- | --- | --- | --- | --- | --- | --- | --- |
| HSHI | 0.049 |  |  |  |  |  |  |  |  |  |  |  |  |  |  |  |  |  |  |  |  |
| HBRE | 0.040 | 0.048 |  |  |  |  |  |  |  |  |  |  |  |  |  |  |  |  |  |  |  |
| HSIE | 0.083 | 0.104 | 0.045 |  |  |  |  |  |  |  |  |  |  |  |  |  |  |  |  |  |  |
| HSEE | 0.076 | 0.094 | 0.045 | 0.004 |  |  |  |  |  |  |  |  |  |  |  |  |  |  |  |  |  |
| HSEL | 0.072 | 0.095 | 0.038 | 0.004 | 0.001 |  |  |  |  |  |  |  |  |  |  |  |  |  |  |  |  |
| HCOU | 0.052 | 0.078 | 0.023 | 0.011 | 0.005 | 0.013 |  |  |  |  |  |  |  |  |  |  |  |  |  |  |  |
| HTRI | 0.044 | 0.090 | 0.037 | 0.057 | 0.058 | 0.054 | 0.030 |  |  |  |  |  |  |  |  |  |  |  |  |  |  |
| HDOU | 0.059 | 0.105 | 0.036 | 0.061 | 0.066 | 0.057 | 0.032 | 0.016 |  |  |  |  |  |  |  |  |  |  |  |  |  |
| HELO | 0.054 | 0.106 | 0.041 | 0.074 | 0.078 | 0.068 | 0.052 | 0.019 | 0.018 |  |  |  |  |  |  |  |  |  |  |  |  |
| HAUL | 0.069 | 0.087 | 0.045 | 0.058 | 0.057 | 0.056 | 0.035 | 0.027 | 0.037 | 0.040 |  |  |  |  |  |  |  |  |  |  |  |
| HGOY | 0.046 | 0.074 | 0.029 | 0.063 | 0.061 | 0.058 | 0.033 | 0.011 | 0.017 | 0.002 | 0.024 |  |  |  |  |  |  |  |  |  |  |
| HJET | 0.092 | 0.104 | 0.051 | 0.078 | 0.071 | 0.075 | 0.046 | 0.026 | 0.032 | 0.032 | 0.032 | 0.014 |  |  |  |  |  |  |  |  |  |
| HSTE | 0.059 | 0.099 | 0.035 | 0.078 | 0.075 | 0.067 | 0.049 | 0.008 | 0.013 | 0.013 | 0.015 | 0.009 | 0.001 |  |  |  |  |  |  |  |  |
| HODE | 0.075 | 0.116 | 0.052 | 0.088 | 0.085 | 0.088 | 0.060 | 0.013 | 0.029 | 0.016 | 0.054 | 0.008 | 0.004 | 0.012 |  |  |  |  |  |  |  |
| HAVE | 0.056 | 0.096 | 0.043 | 0.055 | 0.056 | 0.053 | 0.026 | 0.016 | 0.024 | 0.012 | 0.036 | 0.003 | 0.026 | 0.018 | 0.025 |  |  |  |  |  |  |
| HSCO | 0.056 | 0.086 | 0.036 | 0.072 | 0.076 | 0.065 | 0.040 | 0.021 | 0.018 | 0.001 | 0.038 | 0.002 | 0.023 | 0.002 | 0.017 | 0.014 |  |  |  |  |  |
| HBLA | 0.069 | 0.094 | 0.040 | 0.073 | 0.072 | 0.066 | 0.041 | 0.021 | 0.026 | 0.005 | 0.031 | 0.001 | 0.005 | 0.007 | 0.005 | 0.012 | 0.003 |  |  |  |  |
| HALL | 0.095 | 0.151 | 0.098 | 0.151 | 0.149 | 0.146 | 0.112 | 0.099 | 0.084 | 0.095 | 0.126 | 0.083 | 0.134 | 0.111 | 0.101 | 0.099 | 0.081 | 0.097 |  |  |  |
| HGAV | 0.040 | 0.073 | 0.015 | 0.066 | 0.052 | 0.053 | 0.043 | 0.051 | 0.058 | 0.066 | 0.056 | 0.045 | 0.074 | 0.054 | 0.075 | 0.061 | 0.070 | 0.065 | 0.125 |  |  |
| HNIE | 0.078 | 0.108 | 0.049 | 0.094 | 0.086 | 0.073 | 0.085 | 0.093 | 0.087 | 0.082 | 0.081 | 0.069 | 0.100 | 0.076 | 0.104 | 0.089 | 0.089 | 0.082 | 0.156 | 0.028 |  |
| HNIL | 0.062 | 0.097 | 0.042 | 0.092 | 0.088 | 0.078 | 0.080 | 0.068 | 0.072 | 0.072 | 0.068 | 0.054 | 0.096 | 0.062 | 0.093 | 0.084 | 0.074 | 0.076 | 0.126 | 0.011 | 0.037 |

|  | HSPE | HSHI | HBRE | HSIE | HSEE | HSEL | HCOU | HTRI | HDOU | HELO | HAUL | HGOY | HJET | HSTE | HODE | HAVE | HSCO | HBLA | HALL | HGAV | HNIE | HNIL |
| --- | --- | --- | --- | --- | --- | --- | --- | --- | --- | --- | --- | --- | --- | --- | --- | --- | --- | --- | --- | --- | --- | --- |
| RSPE | 0.002 | 0.033 | 0.026 | 0.074 | 0.064 | 0.066 | 0.044 | 0.041 | 0.055 | 0.059 | 0.057 | 0.039 | 0.080 | 0.052 | 0.072 | 0.057 | 0.060 | 0.067 | 0.107 | 0.024 | 0.060 | 0.049 |
| RSHI | 0.037 | 0.002 | 0.056 | 0.111 | 0.098 | 0.101 | 0.080 | 0.090 | 0.110 | 0.108 | 0.088 | 0.079 | 0.114 | 0.105 | 0.122 | 0.096 | 0.094 | 0.101 | 0.150 | 0.071 | 0.110 | 0.098 |
| RBRE | 0.102 | 0.110 | 0.062 | 0.129 | 0.130 | 0.118 | 0.104 | 0.116 | 0.106 | 0.120 | 0.144 | 0.089 | 0.119 | 0.128 | 0.121 | 0.107 | 0.084 | 0.100 | 0.139 | 0.107 | 0.154 | 0.130 |
| RARQ | 0.082 | 0.089 | 0.037 | 0.099 | 0.103 | 0.095 | 0.075 | 0.087 | 0.081 | 0.091 | 0.112 | 0.063 | 0.091 | 0.095 | 0.091 | 0.078 | 0.060 | 0.075 | 0.123 | 0.083 | 0.130 | 0.107 |
| RORN | 0.034 | 0.064 | 0.001 | 0.036 | 0.031 | 0.029 | 0.015 | 0.026 | 0.027 | 0.023 | 0.043 | 0.017 | 0.045 | 0.024 | 0.035 | 0.029 | 0.025 | 0.029 | 0.081 | 0.022 | 0.047 | 0.044 |
| RVIR | 0.040 | 0.078 | 0.010 | 0.024 | 0.023 | 0.015 | 0.011 | 0.041 | 0.036 | 0.027 | 0.044 | 0.028 | 0.061 | 0.030 | 0.051 | 0.039 | 0.038 | 0.039 | 0.095 | 0.019 | 0.035 | 0.029 |
| RSIE | 0.048 | 0.068 | 0.018 | 0.013 | 0.017 | 0.008 | 0.003 | 0.031 | 0.030 | 0.035 | 0.038 | 0.025 | 0.053 | 0.035 | 0.053 | 0.026 | 0.032 | 0.037 | 0.097 | 0.041 | 0.058 | 0.064 |
| RSEE | 0.062 | 0.093 | 0.035 | 0.008 | 0.007 | 0.003 | 0.017 | 0.046 | 0.047 | 0.049 | 0.047 | 0.044 | 0.071 | 0.053 | 0.073 | 0.039 | 0.053 | 0.053 | 0.119 | 0.051 | 0.083 | 0.082 |
| RSEL | 0.056 | 0.083 | 0.022 | 0.010 | 0.010 | 0.006 | 0.008 | 0.029 | 0.031 | 0.039 | 0.040 | 0.031 | 0.051 | 0.039 | 0.054 | 0.032 | 0.037 | 0.040 | 0.102 | 0.041 | 0.069 | 0.066 |
| RCOU | 0.053 | 0.086 | 0.021 | 0.037 | 0.040 | 0.032 | 0.015 | 0.008 | 0.015 | 0.012 | 0.028 | 0.010 | 0.033 | 0.007 | 0.021 | 0.015 | 0.010 | 0.014 | 0.077 | 0.045 | 0.073 | 0.058 |
| RTRI | 0.047 | 0.099 | 0.031 | 0.060 | 0.061 | 0.054 | 0.028 | 0.014 | 0.020 | 0.010 | 0.034 | 0.013 | 0.036 | 0.006 | 0.014 | 0.024 | 0.011 | 0.016 | 0.075 | 0.045 | 0.087 | 0.054 |
| RDOU | 0.051 | 0.087 | 0.028 | 0.058 | 0.057 | 0.051 | 0.033 | 0.007 | 0.006 | 0.012 | 0.037 | 0.007 | 0.019 | 0.003 | 0.011 | 0.019 | 0.013 | 0.009 | 0.095 | 0.042 | 0.075 | 0.053 |
| RELO | 0.059 | 0.094 | 0.049 | 0.070 | 0.067 | 0.072 | 0.043 | 0.005 | 0.027 | 0.013 | 0.037 | 0.004 | 0.017 | 0.012 | 0.008 | 0.013 | 0.016 | 0.010 | 0.089 | 0.061 | 0.097 | 0.071 |
| RAUL | 0.048 | 0.089 | 0.032 | 0.061 | 0.063 | 0.055 | 0.036 | 0.001 | 0.009 | 0.008 | 0.027 | 0.002 | 0.018 | 0.002 | 0.007 | 0.011 | 0.009 | 0.007 | 0.082 | 0.041 | 0.070 | 0.048 |
| RGOY | 0.055 | 0.077 | 0.033 | 0.059 | 0.056 | 0.054 | 0.033 | 0.004 | 0.016 | 0.006 | 0.015 | 0.003 | 0.002 | 0.013 | 0.004 | 0.009 | 0.007 | 0.004 | 0.106 | 0.051 | 0.074 | 0.070 |
| RJET | 0.043 | 0.081 | 0.020 | 0.062 | 0.060 | 0.063 | 0.043 | 0.002 | 0.020 | 0.004 | 0.020 | 0.000 | 0.011 | 0.008 | 0.000 | 0.017 | 0.013 | 0.007 | 0.113 | 0.038 | 0.083 | 0.056 |
| RSTE | 0.056 | 0.086 | 0.028 | 0.056 | 0.071 | 0.052 | 0.051 | 0.008 | 0.011 | 0.002 | 0.020 | 0.006 | 0.007 | 0.016 | 0.011 | 0.011 | 0.011 | 0.012 | 0.073 | 0.051 | 0.094 | 0.060 |
| RODE | 0.093 | 0.121 | 0.056 | 0.083 | 0.085 | 0.071 | 0.049 | 0.023 | 0.034 | 0.016 | 0.046 | 0.009 | 0.002 | 0.010 | 0.005 | 0.024 | 0.009 | 0.001 | 0.108 | 0.083 | 0.111 | 0.097 |
| RAVE | 0.065 | 0.100 | 0.043 | 0.074 | 0.072 | 0.068 | 0.045 | 0.020 | 0.029 | 0.005 | 0.039 | 0.002 | 0.009 | 0.016 | 0.003 | 0.014 | 0.002 | 0.001 | 0.101 | 0.060 | 0.084 | 0.066 |
| RSCO | 0.066 | 0.095 | 0.037 | 0.062 | 0.061 | 0.060 | 0.035 | 0.006 | 0.022 | 0.012 | 0.029 | 0.001 | 0.002 | 0.005 | 0.000 | 0.008 | 0.011 | 0.001 | 0.105 | 0.058 | 0.083 | 0.075 |
| RBLA | 0.063 | 0.085 | 0.032 | 0.063 | 0.060 | 0.058 | 0.037 | 0.012 | 0.020 | 0.011 | 0.031 | 0.004 | 0.005 | 0.007 | 0.001 | 0.015 | 0.011 | 0.002 | 0.111 | 0.052 | 0.082 | 0.075 |
| RALL | 0.063 | 0.111 | 0.077 | 0.129 | 0.125 | 0.124 | 0.088 | 0.068 | 0.062 | 0.084 | 0.095 | 0.053 | 0.106 | 0.085 | 0.077 | 0.070 | 0.064 | 0.077 | 0.020 | 0.092 | 0.129 | 0.098 |
| RDOR | 0.019 | 0.064 | 0.002 | 0.060 | 0.049 | 0.044 | 0.026 | 0.043 | 0.023 | 0.019 | 0.041 | 0.009 | 0.058 | 0.036 | 0.041 | 0.029 | 0.020 | 0.027 | 0.040 | 0.001 | 0.037 | 0.017 |
| RGAR | 0.041 | 0.078 | 0.026 | 0.100 | 0.091 | 0.087 | 0.043 | 0.073 | 0.036 | 0.038 | 0.067 | 0.030 | 0.078 | 0.062 | 0.063 | 0.042 | 0.032 | 0.046 | 0.032 | 0.054 | 0.094 | 0.085 |
| RGAV | 0.053 | 0.093 | 0.026 | 0.076 | 0.072 | 0.063 | 0.061 | 0.071 | 0.061 | 0.064 | 0.070 | 0.049 | 0.087 | 0.052 | 0.078 | 0.078 | 0.069 | 0.070 | 0.120 | 0.005 | 0.033 | 0.004 |
| RNIE | 0.052 | 0.085 | 0.022 | 0.086 | 0.076 | 0.064 | 0.065 | 0.071 | 0.064 | 0.057 | 0.072 | 0.047 | 0.086 | 0.047 | 0.082 | 0.074 | 0.066 | 0.069 | 0.135 | 0.010 | 0.008 | 0.020 |
| RNIL | 0.038 | 0.075 | 0.027 | 0.088 | 0.083 | 0.077 | 0.065 | 0.057 | 0.050 | 0.058 | 0.055 | 0.029 | 0.074 | 0.049 | 0.059 | 0.053 | 0.052 | 0.054 | 0.100 | 0.009 | 0.049 | 0.006 |

|  | RSPE | RSHI | RBRE | RARQ | RORN | RVIR | RSIE | RSEE | RSEL | RCOU | RTRI | RDOU | RELO | RAUL | RGOY | RJET | RSTE | RODE | RAVE | RSCO | RBLA | RALL | RDOR | RGAR | RGAV | RNIE |
| --- | --- | --- | --- | --- | --- | --- | --- | --- | --- | --- | --- | --- | --- | --- | --- | --- | --- | --- | --- | --- | --- | --- | --- | --- | --- | --- |
| RSHI | 0.024 |  |  |  |  |  |  |  |  |  |  |  |  |  |  |  |  |  |  |  |  |  |  |  |  |  |
| RBRE | 0.101 | 0.127 |  |  |  |  |  |  |  |  |  |  |  |  |  |  |  |  |  |  |  |  |  |  |  |  |
| RARQ | 0.082 | 0.105 | 0.003 |  |  |  |  |  |  |  |  |  |  |  |  |  |  |  |  |  |  |  |  |  |  |  |
| RORN | 0.029 | 0.067 | 0.072 | 0.051 |  |  |  |  |  |  |  |  |  |  |  |  |  |  |  |  |  |  |  |  |  |  |
| RVIR | 0.034 | 0.079 | 0.105 | 0.079 | 0.001 |  |  |  |  |  |  |  |  |  |  |  |  |  |  |  |  |  |  |  |  |  |
| RSIE | 0.044 | 0.073 | 0.086 | 0.063 | 0.005 | 0.006 |  |  |  |  |  |  |  |  |  |  |  |  |  |  |  |  |  |  |  |  |
| RSEE | 0.057 | 0.095 | 0.114 | 0.091 | 0.020 | 0.010 | 0.004 |  |  |  |  |  |  |  |  |  |  |  |  |  |  |  |  |  |  |  |
| RSEL | 0.051 | 0.088 | 0.091 | 0.070 | 0.007 | 0.006 | 0.003 | 0.002 |  |  |  |  |  |  |  |  |  |  |  |  |  |  |  |  |  |  |
| RCOU | 0.051 | 0.090 | 0.095 | 0.066 | 0.009 | 0.013 | 0.008 | 0.022 | 0.010 |  |  |  |  |  |  |  |  |  |  |  |  |  |  |  |  |  |
| RTRI | 0.047 | 0.097 | 0.118 | 0.088 | 0.016 | 0.011 | 0.031 | 0.034 | 0.028 | 0.005 |  |  |  |  |  |  |  |  |  |  |  |  |  |  |  |  |
| RDOU | 0.045 | 0.090 | 0.095 | 0.075 | 0.014 | 0.026 | 0.026 | 0.043 | 0.027 | 0.008 | 0.013 |  |  |  |  |  |  |  |  |  |  |  |  |  |  |  |
| RELO | 0.056 | 0.096 | 0.118 | 0.090 | 0.033 | 0.046 | 0.049 | 0.059 | 0.045 | 0.020 | 0.013 | 0.013 |  |  |  |  |  |  |  |  |  |  |  |  |  |  |
| RAUL | 0.042 | 0.093 | 0.100 | 0.071 | 0.018 | 0.028 | 0.027 | 0.045 | 0.029 | 0.004 | 0.007 | 0.006 | 0.003 |  |  |  |  |  |  |  |  |  |  |  |  |  |
| RGOY | 0.048 | 0.084 | 0.109 | 0.082 | 0.019 | 0.031 | 0.026 | 0.045 | 0.030 | 0.008 | 0.010 | 0.001 | 0.003 | 0.003 |  |  |  |  |  |  |  |  |  |  |  |  |
| RJET | 0.038 | 0.079 | 0.115 | 0.080 | 0.016 | 0.023 | 0.033 | 0.048 | 0.033 | 0.010 | 0.004 | 0.003 | 0.008 | 0.003 | 0.010 |  |  |  |  |  |  |  |  |  |  |  |
| RSTE | 0.052 | 0.093 | 0.088 | 0.053 | 0.010 | 0.025 | 0.021 | 0.036 | 0.019 | 0.016 | 0.015 | 0.006 | 0.004 | 0.015 | 0.017 | 0.006 |  |  |  |  |  |  |  |  |  |  |
| RODE | 0.088 | 0.133 | 0.118 | 0.087 | 0.044 | 0.053 | 0.047 | 0.059 | 0.048 | 0.020 | 0.012 | 0.019 | 0.017 | 0.012 | 0.007 | 0.018 | 0.012 |  |  |  |  |  |  |  |  |  |
| RAVE | 0.062 | 0.107 | 0.104 | 0.076 | 0.025 | 0.036 | 0.039 | 0.056 | 0.041 | 0.009 | 0.007 | 0.009 | 0.011 | 0.003 | 0.003 | 0.002 | 0.017 | 0.002 |  |  |  |  |  |  |  |  |
| RSCO | 0.060 | 0.102 | 0.104 | 0.075 | 0.026 | 0.039 | 0.034 | 0.050 | 0.036 | 0.013 | 0.016 | 0.010 | 0.004 | 0.000 | 0.005 | 0.000 | 0.010 | 0.004 | 0.002 |  |  |  |  |  |  |  |
| RBLA | 0.057 | 0.092 | 0.100 | 0.073 | 0.021 | 0.036 | 0.031 | 0.046 | 0.032 | 0.009 | 0.016 | 0.005 | 0.012 | 0.005 | 0.007 | 0.001 | 0.010 | 0.008 | 0.001 | 0.001 |  |  |  |  |  |  |
| RALL | 0.068 | 0.104 | 0.118 | 0.098 | 0.061 | 0.080 | 0.073 | 0.100 | 0.083 | 0.060 | 0.060 | 0.065 | 0.065 | 0.053 | 0.079 | 0.084 | 0.053 | 0.092 | 0.076 | 0.079 | 0.084 |  |  |  |  |  |
| RDOR | 0.016 | 0.062 | 0.083 | 0.062 | 0.005 | 0.010 | 0.011 | 0.030 | 0.016 | 0.006 | 0.009 | 0.015 | 0.035 | 0.015 | 0.025 | 0.019 | 0.014 | 0.050 | 0.025 | 0.031 | 0.026 | 0.039 |  |  |  |  |
| RGAR | 0.046 | 0.076 | 0.097 | 0.074 | 0.025 | 0.034 | 0.041 | 0.058 | 0.045 | 0.032 | 0.032 | 0.049 | 0.052 | 0.039 | 0.048 | 0.051 | 0.038 | 0.072 | 0.051 | 0.055 | 0.049 | 0.036 | 0.009 |  |  |  |
| RGAV | 0.040 | 0.096 | 0.111 | 0.087 | 0.028 | 0.014 | 0.052 | 0.065 | 0.050 | 0.051 | 0.045 | 0.056 | 0.071 | 0.042 | 0.059 | 0.045 | 0.054 | 0.090 | 0.058 | 0.066 | 0.062 | 0.098 | 0.002 | 0.053 |  |  |
| RNIE | 0.030 | 0.091 | 0.143 | 0.114 | 0.028 | 0.018 | 0.048 | 0.069 | 0.057 | 0.056 | 0.048 | 0.058 | 0.076 | 0.051 | 0.061 | 0.065 | 0.078 | 0.089 | 0.060 | 0.067 | 0.068 | 0.104 | 0.031 | 0.081 | 0.015 |  |
| RNIL | 0.024 | 0.071 | 0.113 | 0.091 | 0.027 | 0.018 | 0.050 | 0.071 | 0.050 | 0.037 | 0.025 | 0.035 | 0.046 | 0.030 | 0.042 | 0.030 | 0.036 | 0.084 | 0.040 | 0.049 | 0.046 | 0.071 | 0.019 | 0.055 | 0.004 | 0.044 |

Table S4: Historical and recent *F*_ST_ among target and donor populations that were temporally sampled. Couples of target and donor populations were ordered by decreasing *F*_st_ temporal difference.

| Populations | | *F*_ST_ | |
| --- | --- | --- | --- |
| Target | Donor | Historical | Recent |
| BRE | SPE | 0.040 | 0.101 |
| ODE | SPE | 0.075 | 0.088 |
| COU | GAV | 0.043 | 0.051 |
| SCO | SPE | 0.056 | 0.060 |
| TRI | SPE | 0.044 | 0.047 |
| ELO | SPE | 0.054 | 0.056 |
| GAV | SPE | 0.040 | 0.040 |
| SEL | GAV | 0.053 | 0.050 |
| JET | SPE | 0.059 | 0.052 |
| BLA | SPE | 0.069 | 0.057 |
| SEE | AUL | 0.057 | 0.045 |
| ALL | GAV | 0.125 | 0.098 |
| DOU | SPE | 0.059 | 0.045 |
| ALL | SPE | 0.095 | 0.068 |
| AUL | SPE | 0.069 | 0.042 |
| SEL | AUL | 0.056 | 0.029 |
| SIE | AUL | 0.058 | 0.027 |
| STE | SPE | 0.092 | 0.038 |
| NIL | SPE | 0.062 | 0.024 |
| NIE | SPE | 0.078 | 0.030 |
| COU | AUL | 0.035 | 0.004 |

Table S5: Genetic clustering of populations using Structure with k=7. Local clusters are given in bold.

| River | Scotland | | Upper- Normandy | | Lower- Normandy | | Brittany | | Allier | | Adour | |
| --- | --- | --- | --- | --- | --- | --- | --- | --- | --- | --- | --- | --- |
|  | Historical | Recent | Historical | Recent | Historical | Recent | Historical | Recent | Historical | Recent | Historical | Recent |
| SPE | **0.69** | **0.71** | 0.05 | 0.04 | 0.05 | 0.04 | 0.10 | 0.11 | 0.05 | 0.02 | 0.05 | 0.07 |
| SHI | **0.89** | **0.91** | 0.02 | 0.02 | 0.01 | 0.02 | 0.03 | 0.03 | 0.03 | 0.02 | 0.02 | 0.01 |
| BRE | 0.35 | 0.02 | **0.28** | **0.93** | 0.04 | 0.01 | 0.08 | 0.02 | 0.05 | 0.01 | 0.20 | 0.01 |
| ARQ | - | 0.02 | **-** | **0.89** | - | 0.01 | - | 0.02 | - | 0.01 | - | 0.04 |
| ORN | - | 0.08 | - | 0.10 | **-** | **0.31** | - | 0.20 | - | 0.06 | - | 0.24 |
| VIR | - | 0.05 | - | 0.08 | **-** | **0.46** |  | 0.13 | - | 0.03 | - | 0.25 |
| SIE | 0.01 | 0.03 | 0.02 | 0.04 | **0.88** | **0.63** | 0.06 | 0.18 | 0.01 | 0.02 | 0.02 | 0.10 |
| SEE | 0.01 | 0.01 | 0.02 | 0.02 | **0.88** | **0.83** | 0.07 | 0.10 | 0.01 | 0.02 | 0.01 | 0.02 |
| SEL | 0.03 | 0.02 | 0.02 | 0.03 | **0.86** | **0.68** | 0.05 | 0.20 | 0.01 | 0.03 | 0.02 | 0.05 |
| COU | 0.04 | 0.01 | 0.04 | 0.05 | **0.79** | **0.32** | 0.07 | 0.53 | 0.02 | 0.02 | 0.03 | 0.06 |
| TRI | 0.06 | 0.03 | 0.03 | 0.05 | 0.02 | 0.16 | **0.79** | **0.65** | 0.02 | 0.02 | 0.08 | 0.08 |
| DOU | 0.02 | 0.04 | 0.03 | 0.03 | 0.05 | 0.07 | **0.83** | **0.82** | 0.04 | 0.03 | 0.03 | 0.02 |
| ELO | 0.07 | 0.03 | 0.04 | 0.02 | 0.04 | 0.02 | **0.77** | **0.87** | 0.04 | 0.01 | 0.06 | 0.05 |
| AUL | 0.04 | 0.05 | 0.01 | 0.03 | 0.04 | 0.06 | **0.85** | **0.79** | 0.01 | 0.03 | 0.05 | 0.04 |
| GOY | 0.05 | 0.05 | 0.02 | 0.02 | 0.03 | 0.02 | **0.84** | **0.85** | 0.03 | 0.02 | 0.03 | 0.04 |
| STE | 0.02 | 0.05 | 0.03 | 0.02 | 0.01 | 0.03 | **0.88** | **0.85** | 0.02 | 0.01 | 0.04 | 0.04 |
| JET | 0.02 | 0.02 | 0.01 | 0.02 | 0.01 | 0.02 | **0.92** | **0.87** | 0.02 | 0.03 | 0.02 | 0.02 |
| ODE | 0.02 | 0.01 | 0.01 | 0.03 | 0.02 | 0.02 | **0.92** | **0.90** | 0.02 | 0.03 | 0.01 | 0.02 |
| AVE | 0.02 | 0.02 | 0.02 | 0.02 | 0.03 | 0.02 | **0.89** | **0.89** | 0.02 | 0.02 | 0.02 | 0.03 |
| SCO | 0.02 | 0.01 | 0.03 | 0.02 | 0.04 | 0.02 | **0.88** | **0.92** | 0.01 | 0.02 | 0.03 | 0.01 |
| BLA | 0.02 | 0.02 | 0.02 | 0.03 | 0.02 | 0.06 | **0.91** | **0.86** | 0.01 | 0.02 | 0.02 | 0.03 |
| ALL | 0.01 | 0.01 | 0.02 | 0.01 | 0.01 | 0.01 | 0.02 | 0.03 | **0.94** | **0.91** | 0.01 | 0.02 |
| DOR | - | 0.07 | - | 0.04 | - | 0.04 | - | 0.06 | - | 0.37 | - | 0.41 |
| GAR | - | 0.10 | - | 0.04 | - | 0.05 | - | 0.09 | - | 0.54 | - | 0.19 |
| GAV | 0.06 | 0.04 | 0.04 | 0.06 | 0.02 | 0.05 | 0.04 | 0.09 | 0.01 | 0.03 | **0.84** | **0.74** |
| NIE | 0.02 | 0.02 | 0.01 | 0.02 | 0.02 | 0.01 | 0.02 | 0.02 | 0.01 | 0.01 | **0.92** | **0.92** |
| NIL | 0.09 | 0.07 | 0.08 | 0.02 | 0.03 | 0.04 | 0.07 | 0.09 | 0.03 | 0.05 | **0.70** | **0.73** |

Table S6: Admixture of each sample determined using Structure with separate analyses for estimating admixture in Upper-Normandy, k=3 (I), in Lower-Normandy, k=4 (II), in Brittany, k=2 (III) and in Allier-Gironde-Adour, k=4 (IV). Local clusters given in bold, stocked samples highlighted in grey.

| Analysis | River | Scotland | | Upper- Normandy | | Lower- Normandy | | Brittany | | Allier | | Adour | |
| --- | --- | --- | --- | --- | --- | --- | --- | --- | --- | --- | --- | --- | --- |
|  |  | Historical | Recent | Historical | Recent | Historical | Recent | Historical | Recent | Historical | Recent | Historical | Recent |
| I | BRE | 0.60 | 0.02 | **0.40** | **0.98** |  |  |  |  |  |  |  |  |
|  | ARQ | - | 0.04 | **-** | **0.96** |  |  |  |  |  |  |  |  |
| II | ORN | - | 0.09 |  |  | **-** | **0.38** | - | 0.26 |  |  | - | 0.27 |
|  | VIR | - | 0.08 |  |  | **-** | **0.50** | - | 0.14 |  |  | - | 0.28 |
|  | SIE | 0.01 | 0.03 |  |  | **0.89** | **0.68** | 0.07 | 0.18 |  |  | 0.03 | 0.11 |
|  | SEE | 0.01 | 0.01 |  |  | **0.91** | **0.86** | 0.06 | 0.10 |  |  | 0.02 | 0.03 |
|  | SEL | 0.04 | 0.03 |  |  | **0.89** | **0.69** | 0.04 | 0.22 |  |  | 0.03 | 0.06 |
|  | COU | 0.05 | 0.02 |  |  | **0.82** | **0.32** | 0.07 | 0.59 |  |  | 0.06 | 0.07 |
| III | TRI | 0.25 | 0.21 |  |  |  |  | **0.75** | **0.79** |  |  |  |  |
|  | DOU | 0.12 | 0.11 |  |  |  |  | **0.88** | **0.89** |  |  |  |  |
|  | ELO | 0.11 | 0.07 |  |  |  |  | **0.89** | **0.93** |  |  |  |  |
|  | AUL | 0.18 | 0.14 |  |  |  |  | **0.82** | **0.86** |  |  |  |  |
|  | GOY | 0.10 | 0.11 |  |  |  |  | **0.90** | **0.89** |  |  |  |  |
|  | STE | 0.03 | 0.08 |  |  |  |  | **0.97** | **0.92** |  |  |  |  |
|  | JET | 0.04 | 0.06 |  |  |  |  | **0.96** | **0.94** |  |  |  |  |
|  | ODE | 0.03 | 0.04 |  |  |  |  | **0.97** | **0.96** |  |  |  |  |
|  | AVE | 0.05 | 0.03 |  |  |  |  | **0.95** | **0.97** |  |  |  |  |
|  | SCO | 0.03 | 0.02 |  |  |  |  | **0.97** | **0.98** |  |  |  |  |
|  | BLA | 0.05 | 0.05 |  |  |  |  | **0.95** | **0.95** |  |  |  |  |
| IV | ALL | 0.01 | 0.03 |  |  |  |  |  |  | **0.98** | **0.94** | 0.01 | 0.03 |
|  | DOR | - | 0.09 |  |  |  |  |  |  | - | 0.42 | - | 0.49 |
|  | GAR | - | 0.17 |  |  |  |  |  |  | - | 0.61 | - | 0.22 |
|  | GAV | 0.07 | 0.07 |  |  |  |  |  |  | 0.02 | 0.04 | **0.91** | **0.89** |
|  | NIE | 0.02 | 0.03 |  |  |  |  |  |  | 0.01 | 0.02 | **0.97** | **0.95** |
|  | NIL | 0.13 | 0.10 |  |  |  |  |  |  | 0.06 | 0.07 | **0.81** | **0.83** |
